# The maize leaf lipidome shows multilevel genetic control and high predictive value for agronomic traits

**DOI:** 10.1038/srep02479

**Published:** 2013-08-21

**Authors:** Christian Riedelsheimer, Yariv Brotman, Michaël Méret, Albrecht E. Melchinger, Lothar Willmitzer

**Affiliations:** 1 Institute of Plant Breeding, Seed Science and Population Genetics, University of Hohenheim, Fruwirthstr. 21, 70593 Stuttgart, Germany; 2 Max-Planck Institute of Molecular Plant Physiology, Am Mühlenberg 1, 14476 Potsdam, Germany; 3 These authors contributed equally to this work.

## Abstract

Although the plant lipidome show an enormous level of structural and functional diversity, our knowledge about its genetic control and its connection to whole-plant phenotypes is very limited. Here, we profiled 563 lipid species with UPLC-FT-MS in 289 field-grown inbred lines genotyped with 56,110 SNPs. Genome-wide association study identified 174 associations for 76 lipids explaining up to 31.4% of the genetic variance (*P*-value 8.4 × 10^−18^). Candidate genes were found for lipid synthesis, breakdown, transfer, and protection against peroxidation. The detected SNP-lipid associations could be grouped into associations with 1) individual lipids, 2) lipids from one biochemical class, and 3) lipids from several classes, suggesting a multilevel genetic control architecture. We further found a strong connection between the lipidome and agronomic traits in field-evaluated hybrid progeny. A cross-validated prediction model yielded correlations of up to 0.78 suggesting that the lipidome accurately predicts agronomic traits relevant in hybrid maize breeding.

Living organisms produce a plethora of chemically distinct lipid species, commonly referred to as the lipidome^1^. Lipids are fascinating for their chemical diversity which is especially enormous in plant domain due to the high plasticity of their biosynthetic machinery^2^. In addition, lipids are involved in various cellular processes including cell integrity, membrane formation and scaffolding for membrane proteins, energy storage, and cell signaling^3^.Lipid composition has been demonstrated to change with environmental conditions, e.g. under various abiotic stresses, and has actually been demonstrated to serve as an indicator for the respective stress^4^. Recent progress in mass spectrometry allows measuring simultaneously hundreds of chemically different lipid species in a single sample of plant tissue, which though impressive, covers only a part of the complexity of the lipidome^5^,^6^,^7^,^8^,^9^.

Although in *Arabidopsis thaliana*, most of the biochemical steps involved in lipid biosynthesis are known and the key genes have been identified, the regulation of the processes that results in the final lipid composition is only weakly understood. Quantitative genetic approaches have been followed more recently in *Arabidopsis thaliana*^10^ and maize^11^ to identify loci involved in storage oil formation; however this analysis was limited to one class of lipids, *i.e.* triacylglycerides. Recently, we have described results obtained for mapping primary metabolites in maize^12^ via genome-wide association (GWA) mapping. The GWA mapping approach relies on ancestral linkage disequilibrium (LD) in a diverse population and can yield a mapping resolution up to the single nucleotide level.

Using the same mapping population we here extend this study to identify numerous loci involved in lipid metabolism. Using UPLC-FT-MS, we measured 563 distinct leaf lipids in 289 diverse maize inbred lines genotyped with 56,110 SNPs. In addition we asked the question whether or not lipid profiles can be used for predicting general combining ability (GCA) of the parent lines reflecting the average performance of their hybrid progeny as previously successfully shown for primary metabolites^13^. GCA was estimated for four traits by crossing 285 lines from the Dent heterotic pool with two single-cross testers from the Flint heterotic pool and evaluating their testcross progeny in three locations over two years.

## Results

### Lipid profiling

**Figure 1:**
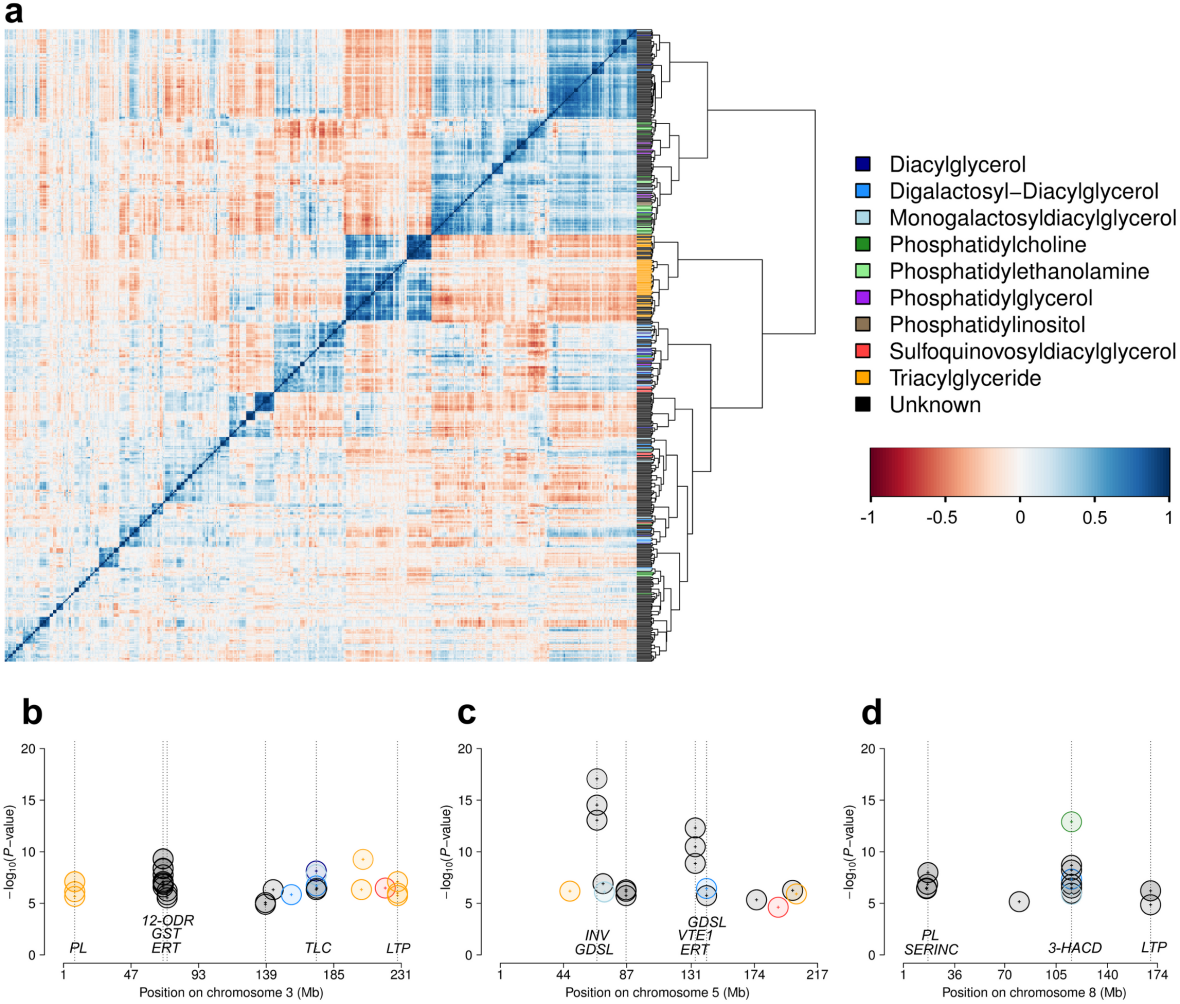
Correlation and colocalization pattern of lipids. (a) Pairwise Pearson correlations (r) among all 563 measured lipids. Lipids are ordered using Ward clustering on pairwise dissimilarity, calculated as 1 – r. The cluster on the right is colored according to the chemical class of the lipids. (b–d) Colocalization of GWA mapping hits on chromosomes 3,5, and 8. Circles refer to –log_10_(*P*-value) of SNP-lipid associations which were significant with FDR ≤ 0.025. Dotted lines indicate genomic positions with association signals of multiple lipids. Candidate genes with known relationships to lipid metabolism or protection against peroxidation are shown at the bottom. *PL*, phospholipase; *12-ODR*, 12-oxophytodienoate reductase; *GST*, gluthation-S-transferase; *ERT*, ethylene-responsive transcription factor; *TLC*, TRAM/LAG1/CLN8 (TLC) lipid-sensing domain containing protein; *LTP*, lipid transfer protein; *INV*, invertase; *GDSL*, GDSL esterase/lipase; *VTE1*, tocopherol cyclase; *SERINC*, serinc-domain containing sphingolipid biosynthesis protein; *3-HACD*, 3-hydroxyacyl-CoA dehydratase. Details of all association hits can be found in Supplementary Table S2 and candidate genes in Table 1. Colocalizations on all other chromosomes are presented in Supplementary Fig. S2.

Using ultra-performance liquid chromatography separation coupled to a high-resolution Fourier transform mass spectrometer (UPLC-FT-MS), 563 distinct leaf lipids were measured in the 289 maize inbred lines from the diversity panel of worldwide sources (Supplementary Table S1). It is clear that these 563 species scratch only the surface of the lipidome, however, as demonstrated below, they already allowed to identify a number of loci/regions in the maize chromosome associated with their abundance in maize leaves. From the mass spectra of the detected compounds, 16 could be annotated to be diacylglycerols (DAG), 15 as digalactosyl-diacylglycerols (DGDG), 23 as monogalactosyl-diacylglycerols (MGDG), 24 as phosphatitylcholines (PC), 11 as phosphatidylethanolamines (PE), six as phosphatidylglycerols (PG), three as phosphatitylinositols (PI), nine as sulfoquinovosyldiacyl-glycerols (SQDG), and 48 as triacylglycerides (TAG). The remaining lipids could not be annotated at present, however, specifically for lipids showing a strong association with gene loci, efforts are presently undertaken to clarify their structure. Lipid concentrations showed an average repeatability (w^2^) of 0.66 (Supplementary Fig. S1), with a total of 205 compounds (36.4%) having w^2^ > 0.8. The lipids showed a correlation pattern with intense substructuring (Fig. 1a). Notably, all annotated TAGs clustered together into one highly positively correlated group.

### Genome-wide association mapping

**Figure 2:**
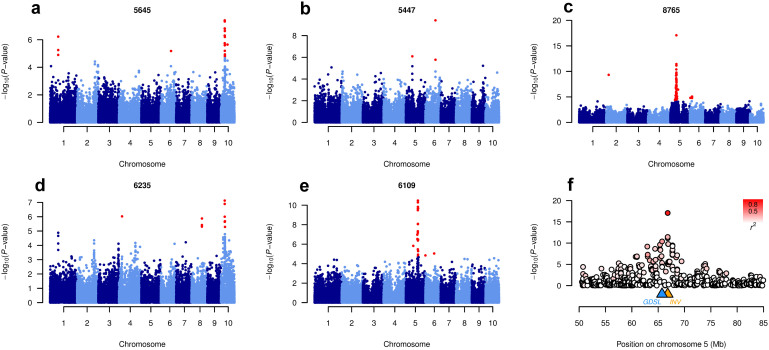
Five examples of GWA mapping results. (a–e) Manhattan plots for lipids 5645, 5447, 8765, 6235, and 6109. *P*-values obtained from a **Q**_10_ + **K** mixed model analysis are shown on a –log_10_-scale and colored in red if significant with FDR ≤ 0.025. (f) Regional association plot for lipid 8765. The positions of the candiate genes GDSL esterlase/lipase and invertase (*INV*) are shown as triangles. Level of linkage disequilibrium (r^2^) with the top hit SNP is shown in red color.

Using the **Q**_10_ + **K** mixed model and a FDR threshold of 2.5%, we detected 174 SNP-lipid associations for 76 lipids, explaining up to 31.6% of the genetic variance (Supplementary Table S2 and Fig. 2). A total of 23 lipids showed at least one signal explaining more than 15% of the genetic variance. For the lipids with significant association signals, the average inflation factor λ was 1.14. From the annotated lipids, most significant hits were found for TAG and MGDG (Table S2). Associations were found on all chromosomes, with an intense co-localization of the same signal positions for groups of lipids (Fig. 1b–d and Supplementary Fig. S2).

The strongest SNP-lipid associations were found for three lipid species with unknown chemical structure, which were highly correlated with each other (0.96 < r < 0.99). All three showed their strongest associations (*P*-values ranging from 8.8 × 10^−14^ to 8.4 × 10^−18^) at position 66.8 Mb on chromosome 5, nearby (1.07 Mb) a lipolytic GDSL esterase/lipase and very close (4.5 kb) to an invertase gene (Fig. 2c,f ).

**Figure 3:**
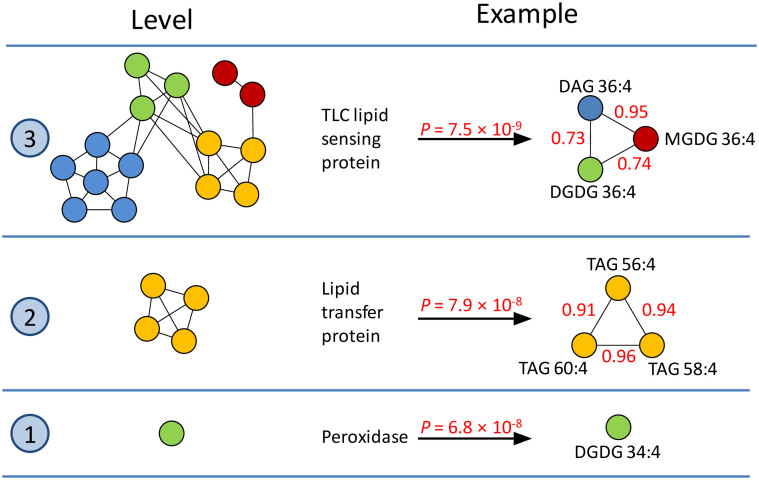
Examples for the control levels 1–3 which have been assigned to the detected candidate genes. A TLC lipid sensing protein was detected for lipids with the same fatty acid residual but different backbones and hence different chemical classes. A lipid transporter was associated with three strongly correlated TAGs with 56 to 60 C atomes. In contrast, a peroxidase was uniquely found for a single lipid species. Different chemical classes are shown as different colors. Red values on the edges correspond to pairwise correlations. The shown *P*-value reflects the lowest value for all associations with the candidate gene.

Candidate genes involved in lipid synthesis, transfer, breakdown, signaling and protection against peroxidation were identified for many detected associations (Table 1). Candidate genes were grouped into three hierarchy levels depending on the biochemical composition of the compound(s) for which the associations were detected (Fig. 3). Level 1 candidate genes correspond to associations uniquely found for one lipid species. Level 2 candidate genes represent associations specific to several lipids from one chemical class. Finally, level 3 candidate genes correspond to associations found for lipids from multiple chemical classes.

Level 1 candidate genes include a peroxidase on chromosome 2 which was uniquely associated with DGDG 34:4 and a phospholipase, which was found for an unknown lipid (ID 6151).

The biochemically closely related TAGs 56:4, 58:4, and 60:4 showed two associations leading to level 2 candidate genes. The first association was found at the beginning of chromosome 3 at 8.6 Mb, very close (268.3 kb) to a phospholipase, and the second at the end of the chromosome at 228.4 Mb, nearby a lipid transporter.

Examples for level 3 candidate genes include amongst others a sphingosine kinase, found 294.5 kb away from an association at position 33.56 Mb on chromosome 4, for which one PG, one MGDG, and three unknown lipids mapped together. One PG, one MGDG, one SQDG, and nine unknown lipids mapped to a region from 41.4 to 42.4 Mb on chromosome 10, harboring two genes, the products of which have a high similarity (Protein-Protein BLAST E-values 4 × 10^−64^ and 5 × 10^−86^) to a fatty acid desaturase in *Arabidopsis*. Nine out of these 13 lipids also showed a common association on chromosome 1 nearby (538.1 kb) a lipid transfer protein. In addition, eight lipids from three chemical classes mapped to the same position at 115.5 Mb on chromosome 8, in close vicinity (4.9 kb) of a 3-hydroxyacyl-CoA dehydratase, a key component in the elongase complex for synthesizing very-long-chained fatty acids^14^. Finally, one DGDG, one MGDG, and one DAG, all sharing the same fatty acid residual (36:4), mapped besides two unknown lipids to the same location at 173.1 Mb on chromosome 3, close (81.5 kb) to a protein containing a TLC (TRAM/LAG1/CLN8) lipid-sensing domain (Fig. 3).

Other candidate genes with known relationship to lipid metabolism include ethylene-responsive transcription factors, tocopherol cyclase, gluthation-S-transferase, as well as 12-oxophytodienoate reductase, which participates in the conversion of linolenic acid into jasmonic acid, a fatty acid derivate acting as an important plant hormone.

### Whole-lipidome prediction of general combining ability

**Figure 4:**
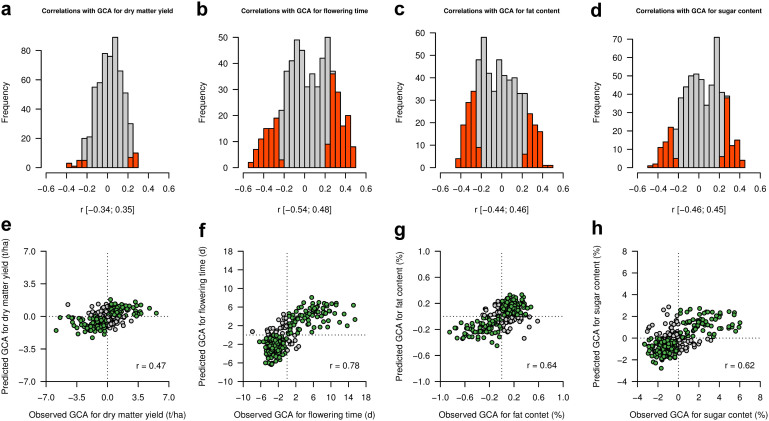
Whole-lipidome prediction of GCA for dry matter yield, flowering time, fat and sugar contents. (a–d) Histograms showing Pearson correlations between individual lipids and GCA values. Correlations significant at a Bonferroni-corrected 5% threshold (*P*-value < 8.9 × 10^−5^) are colored in red. (e–h) Observed *vs*. predicted GCA values using whole-lipidome prediction. Average values obtained from a 20 times repeated 5-fold cross-validation are presented. Genotypes with a correct classification of GCA values are colored in green.

Correlations between individual lipids with GCA values ranged from –0.54 to 0.48 (Fig. 4a–d). To predict GCA with the lipidome, we applied a statistical prediction model that assumes that all lipids have small, normally distributed effects associated with the target trait.

Using a 20-times repeated 5-fold cross-validation scheme, the correlations between observed and predicted GCA values ranged from 0.47 for dry matter yield to 0.78 for flowering time, with quality traits being better predictable than dry matter yield (Fig. 4e–h). The predictive ability matched with the number of Bonferroni-corrected significant correlations with GCA (Fig. 4A–D, red portion), which was highest for flowering time (190) and considerably higher for sugar (130) and fat content (162) compared to dry matter yield (31).

## Discussion

The composition of the detected, genetically co-regulated modules in the lipidome provided hints about the hierarchical position of the underlying controlling genes. Whereas level 1 suggests genes operating at the most elementary level for modifying or controlling individual lipids, level 2 candidate genes are assumed to control sets of biochemically closely related lipids of one chemical class. On top, associations found for large groups of structurally distinct lipids (level 3) suggest underlying causal genes regulating the lipidome at a higher hierarchical level.

Currently, incomplete annotations limit our ability to distinguish perfectly the level 3 from level 2 candidate genes. Nevertheless, the suggested hierarchy levels largely corresponded with the specificity of the associated candidate genes. For example, the lipid signaling molecule sphingosine-1-phosphate has recently received great attention because of its involvement in many physiological processes in yeast, mammals, and plants^15^,^16^, suggesting a higher hierarchical control position conserved over species. It was therefore not surprising that the underlying gene sphingosine kinase was associated with lipids from multiple chemical classes. Likewise, the conserved TRAM/LAG1/CLN8 (TLC) lipid-sensing domain, reported to be involved in several lipid processes^17^, was found in a product of a level 3 candidate gene associated with three lipids with different backbones but the same fatty acid residual (Fig. 3). Similarly, the level 3 ethylene-responsive transcription factor was described to control different lipid pathways in *Arabidopsis*^18^ and barley^19^. On the contrary, lipid transporters are known to be more substrate specific^20^, especially if covalently modified^21^. This makes the detected level 2 triacylglyceride specific transporter a promising candidate for further functional investigations.

Two candidate genes, GRMZM2G084264, GRMZM2G073258 (Table 1), show high homology to the three members (SHN1, SHN2 and SHN3) of the *Arabidopsis* SHN transcription factors which are involved in the regulation of a variety of lipid metabolism processes including lipid transport, fatty acid elongation, and the synthesis of wax, suberin, and cutin^18^,^22^,^23^. The gene GRMZM2G176175 shows high homology (Protein-Protein BLAST E-values 8 × 10-33) to the gene WRI1 that belongs to the protein family of the AP2/EREBP transcription factors in *Arabidopsis* which have been demonstrated to be involved in fatty acid synthesis and triacylglycerol biosynthesis processes^24^,^25^,^26^.

On chromosome 4, a candidate gene showed a high similarity (Protein-Protein BLAST E-value 1 × 10^−12^) to *CER2*, a gene studied in detail in *Arabidopsis*, which is required for the elongation of fatty acids of exceptional length^27^. Our results also propose that protection against peroxidation needs to be particularly considered. In addition to several glutathion-S-transferases^28^, we also detected an invertase gene that reduces membrane lipid peroxidation during chilling temperatures if artificially expressed in the apoplast of potato^29^.

Lipid synthesis occurs via sequential addition of two-carbon units derived from acetyl CoA. which is nicely reflected in the mapping of biochemically closely related lipids from specific lipid classes such as TAGs 56:4, 58:4, and 60:4 to just one locus (Fig. 1b). The DAG moiety has been proposed as the major pathway for TAG synthesis in excised linseed (*Linum usitatissimum*) and soybean (*Glycine max*) cotyledons^30^. The DAG moieties derived from phospholipid hydrolysis are used in galactolipid biosynthesis^31^. We identified a phospholipase in close vicinity to the respective SNP which might carry the causal variant of this association (Fig. 1b). It is obvious that like in any association study, the loci identified remain candidate genes, which need further validation via e.g. reverse genetics approaches for final annotation.

The rich functional diversity of the lipidome and its association to a diversity of biological processes suggests that it might be used for the prediction of phenotypic performance. We tested this hypothesis by predicting agronomic relevant traits evaluated in hybrid progeny in multi-environment field trials. The high prediction accuracy confirmed by cross-validation showed that such an approach is very promising. In addition, the higher absolute correlation coefficients and cross-validated predictions for flowering time, and sugar and fat content compared to dry matter yield indicated that the leaf lipidome is in general more closely connected to quality or developmental traits than to biomass.

In conclusion, our study demonstrates that lipidomics is not only a powerful tool for shedding light on the genetic control of the lipid metabolism in crop plants, but it also provides access to a novel layer of biochemical information exploitable for predicting agronomic traits relevant in hybrid maize breeding.

## Methods

### Genetic material

The genetic material consisted of (i) a previously described diversity panel of 285 Dent inbred lines^12^ with additional four genotypes as checks, and (ii) the 570 testcrosses produced by crossing the 285 inbred lines to two single cross European Flint testers^13^.

### Genotyping

Genotyping was performed using the Illumina SNP chip Maize-SNP50 (Illumina Inc.) containing 56,110 unique SNPs^32^. A quality preprocessing was done by applying the following criteria: (i) call rate above 0.95, (ii) unique allele assignment for the 22 replicated checks of genotype B73, (iii) minor allele frequency greater than 2.5%, and (iv) no more than three heterozygous genotypes. A total of 37,282 SNPs met these criteria. Five genotypes with a residual heterozygosity above 5% were excluded. The chromosomal positions of the SNPs refer to the B73 reference genome (B73 RefGen_v1). Candidate genes were taken from the B73 filtered gene set (release 4a.53).

### Lipid profiling

Inbred lines in field trials were subject to lipid profiling. Genotypes were divided into three maturity groups and randomized as three adjacent 20 × 5 α-lattice designs with two replications and planted in two-row plots. Leaf samples of ≈ 5 cm were cut from the middle part of the fully developed third leaf of 10 plants per plot, bulked, and immediately frozen using dry ice. The five plots of every incomplete block were sampled within a period of 15 s to minimize within-block error due to metabolic changes over time. The 50 samples from 10 randomly chosen blocks of one field replication of one maturity group were subsequently processed together as one batch. With this blocking structure, we could account for systematic shifts among batches while keeping the field randomization intact. Samples on batches were lyophilized and stored for the subsequent analysis. Samples were processed using ultra-performance liquid chromatography coupled with Fourier transform mass spectrometry (UPLC-FT-MS, ref. 6) on a C8 reverse phase column coupled with an Exactive mass spectrometer (Thermo-Fisher, http://www.thermofisher.com) in positive ionization mode. Processing of chromatograms, peak detection and integration were performed using REFINER MS® 5.3 (GeneData, http://www.genedata.com). Processing of mass spectrometry data included the removal of the fragmentation information, isotopic peaks, as well as chemical noise. Obtained features (*m/z* at a certain retention time) were queried against an in-house lipid database for further annotation. Remaining unknown features were crossed checked with a different, on-line database using an in-house developed database search tool (Golm Biochemical Space, GOBIOSPACE) for putative annotation.

### Multi environment trials of testcrosses

The 570 testcrosses were used to estimate GCA values in multi-environment field trials in three agroecologically diverse locations for two years (2008 and 2009) in Germany, as described previously^13^.Dry matter yield of whole-plant biomass (t/ha), and female flowering (d) were measured per field plot. Fat and sugar contents (%) were measured in 1 kg of harvested plant material from each plot using calibrated near-infrared spectroscopy (NIRS^33^).

### Phenotypic analysis

Mass intensities were scaled by the median of each sample. Linear mixed model analysis was used to obtain least squares means of lipid levels and GCA values. The model for the lipids was G + M + M·R + M·R·A + M·R·A·B, with the following effects: genotype (G), trial of maturity group (M), field replication (R), batch (A), and block (B). Random effects are underlined. To achieve homoscedasticity of the model residuals for the lipids, the flexible Box-Cox power transformation was applied. For each lipid, the optimum transformation parameter was determined using the maximum likelihood method described previously^34^. Repeatabilities were calculated as w^2^ = σ^2^_g_/(σ^2^_g_ + σ^2^_e_/2) where σ^2^_g_ is the genotypic variance estimated by REML, by setting factor G as random and σ^2^_e_ is the residual variance. Following established analysis, the testcross genotype was decomposed as G = GCA + T + SCA, where GCA is the main effect of the line, T is the main effect of the tester genotype, and SCA (specific combining ability) is the line × tester interaction. Including an effect for the environment (E), defined as the year × location combination, the final model for estimating GCA was therefore GCA + T + SCA + GCA·E + T·E + SCA·E + E + E·M + E·M·R + E·M·R·B.

### Genome-wide association mapping

The GWA mapping with plant populations is often hampered by population structure and cryptic relatedness, resulting in spurious associations^35^. However, powerful techniques are available to decouple genetic associations from confounding factors^36^. We used a mixed model approach which corrects simultaneously for the main directions of population substructure by regression on the first ten principal components (**Q_10_**), as well as pairwise relatedness by using an allele-sharing kinship matrix **K** as covariance matrix for the random genotype effects^37^. For an appropriate significance threshold for SNP–trait associations, we controlled the false discovery rate (FDR), as previously suggested for GWA mapping^38^. Genome-wide inflation factors (λ) were calculated as the regression coefficient in the QQ plot with a zero intercept. The GWA models were fitted using the maximum likelihood implementation in the function polygenic of GenABEL^39^. *P*-values were obtained with the 1 degree of freedom fast association score test–based analysis (FASTA), implemented in the function mmscore of GenABEL. *P*-values were transformed to q-values and regarded significant if smaller than 0.025 to control for a false discovery rate (FDR) of 2.5%. The proportion of genetic variance explained by a certain SNP was calculated as ρ = R^2^_LR_/w^2^ using the likelihood-ratio (LR) statistic R^2^_LR_ = 1 – exp(–LR/n) with LR = 2 × log(L_SNP_/L_0_), where L_0_ is the maximum likelihood of the baseline **Q_10_ + K** model without considering the SNP, L_SNP_ is the maximum likelihood of the full **Q_10_ + K** model including the SNP as cofactor, and n is the number of genotypes^40^.

### Whole-lipidome prediction of GCA

We applied ridge regression BLUP (RR-BLUP^41^), for prediction of GCA values using all lipids as predictor variables. The model makes two assumptions: (i) normally distributed predictor effects, and (ii) normally distributed errors. Validation was performed using a 20 times repeated 5-fold cross validation. The data set was divided into five disjoint subsets of genotypes, where one subset was left out for validation. The other four subsets were used as the training population to estimate the model parameters for predicting the observations of the left-out genotypes in the validation population. In each of the five rounds, the Pearson correlation between observed and predicted GCA values was calculated. Twenty different randomizations for assigning the genotypes to five different subsets were used to yield 100 cross-validation runs. The predictive ability was then calculated as the Pearson correlation between the observed and predicted GCA values averaged over all cross-validation runs.

## Author Contributions

C.R. performed leaf sampling, phenotypic analysis, genome-wide association and whole-lipidome prediction. C.R. and Y.B. carried out the annotation of the candidate genes. M.M. and Y.B. performed lipid profiling and M.M. accomplished the chemical annotation. C.R. and Y.B. wrote the manuscript. L.W. and A.E.M. designed the experiments and supervised the research.

## Supplementary Material

Supplementary Information Supplementary information

## Figures and Tables

**Table 1:** Details of candidate genes with known relationships to lipid metabolism and protection against peroxidation. Positions refer to the B73 reference genome RefGen_v1

Chr.	Position	Candidate gene	Distance to candidate gene (kb)	Product of candidate gene	Control level
1	90435505	GRMZM2G079568	538.1	Lipid transfer protein	2
2	106409512	GRMZM2G067096	0.0	Peroxidase	1
	27559497	GRMZM2G138701	11.0	C2 Ca^2+^/lipid-binding domain-containing phosphoribosyltransferase	2
		GRMZM2G178693	229.4	Aquaporin	2
3	8610977	GRMZM2G353444	268.3	Phospholipase	2
	68792482	GRMZM2G068947	286.9	12-oxophytodienoate reductase	2
		GRMZM2G146887	83.5	Gluthation-S-transferase	2
		GRMZM2G146913	97.3	Gluthation-S-transferase	2
		GRMZM2G073258	430.6	Ethylene-responsive transcription factor	2
	173118447	GRMZM2G077279	81.5	TRAM/LAG1/CLN8 (TLC) lipid-sensing domain containing protein	3
	204973260	GRMZM2G434541	71.9	Gluthation-S-transferase	1
	228429469	GRMZM2G010868	970.5	Lipid transfer protein	2
4	33567688	GRMZM2G088549	294.5	Sphingosine kinase	3
	85049647	GRMZM2G032276	556.2	*CER2* fatty acid elongase-like protein	2
5	66804095	GRMZM2G089836	4.5	Vacuolar invertase	2
		GRMZM2G374475	268.3	GDSL esterase/lipase	2
	133758422	GRMZM2G073258	430.6	Ethylene-responsive transcription factor	2
		GRMZM2G084264	312.1	Ethylene-responsive transcription factor	2
		GRMZM2G009785	970.1	Tocopherol cyclase	3
	141450112	GRMZM2G159759	263.1	GDSL esterase/lipase	3
6	23858424	GRMZM2G035502	6.0	Gluthation-S-transferase	2
	98818869	GRMZM2G129642	86.1	C2 Ca^2+^/lipid-binding domain-containing phosphoribosyltransferase	2
		GRMZM2G100864	206.4	C2 Ca^2+^/lipid-binding domain-containing phosphoribosyltransferase	2
	111523070	GRMZM2G084264	312.1	Ethylene-responsive transcription factor	3
		GRMZM2G004276	531.6	Triacylglycerol lipase	3
7	23152493	GRMZM2G176175	1149.3	Ethylene-responsive transcription factor	2
	134997749	GRMZM2G071015	164.0	C2 Ca^2+^/lipid-binding and GRAM domain-containing protein	1
8	17434337	GRMZM2G061969	0.0	Phospholipase	1
	17706101	GRMZM2G088356	77.6	Serinc-domain containing serine and sphingolipid biosynthesis protein	2
	115491648	GRMZM2G035202	4.9	3-hydroxyacyl-CoA dehydratase	2
	169339892	GRMZM2G379035	408.3	Lipid transfer protein	2
10	41583258	GRMZM2G175401	878.2	Fatty acid desaturase A	3
		GRMZM2G097509	1095.3	Fatty acid desaturase A	3
	72379858	GRMZM2G022359	506.5	Ethylene-responsive transcription factor	2

## References

[b1] Mutch D. M., Fauconnot L., Grigorow M. & Fay L. B. Putting the ‘Ome' in lipid metabolism. Biotechnol. Annu. Rev. 12, 67–84 (2006). 17045192 10.1016/S1387-2656(06)12003-7

[b2] Broun P., Shanklin J., Whittle E. & Somerville C. Catalytic plasticity of fatty acid modification enzymes underlying chemical diversity of plant lipids. Science 282, 1315–1317 (1998). 9812895 10.1126/science.282.5392.1315

[b3] Brown H. A. & Murphy R. C. Working toward an exegesis for lipids in biology. Nat. Chem. Biol. 9, 602–606 (2009). 19690530 10.1038/nchembio0909-602 PMC3785062

[b4] Vu H. S. *et al.* Direct infusion mass spectrometry of oxylipin-containing Arabidopsis membrane lipids reveals varied patterns in different stress responses. Plant Physiol. 158, 324–339 (2012). 22086419 10.1104/pp.111.190280 PMC3252110

[b5] Welti R. & Wang X. Lipid species profiling: a high-throughput approach to identify lipid compositional changes and determine the function of genes involved in lipid metabolism and signaling. Curr. Opin. Plant Biol. 7, 337–344 (2004). 15134756 10.1016/j.pbi.2004.03.011

[b6] Hummel J. *et al.* Ultra Performance Liquid Chromatography and High Resolution Mass Spectrometry for the Analysis of Plant Lipids. Front. Plant Sci. 2, 1–17 (2011). 22629264 10.3389/fpls.2011.00054 PMC3355513

[b7] Horn P. J. & Chapman K. D. Lipidomics in tissues, cells and subcellular compartments. Plant J. 70, 69–80 (2012). 22117762 10.1111/j.1365-313X.2011.04868.x

[b8] Wenk M. R. Lipidomics: new tools and applications. Cell 143, 888-895 (2010). 21145456 10.1016/j.cell.2010.11.033

[b9] Wenk M. R. The emerging field of lipidomics. Nat. Rev. Drug Discov. 4, 594–610 (2005). 16052242 10.1038/nrd1776

[b10] O'Neill C. M. *et al.* Towards the genetic architecture of seed lipid biosynthesis and accumulation in Arabidopsis thaliana. Heredity (Edinb) 108, 115–123 (2012). 21731053 10.1038/hdy.2011.54 PMC3262871

[b11] Li H. *et al.* Genome-wide association study dissects the genetic architecture of oil biosynthesis in maize kernels. Nat. Genet. 45, 43–50 (2013). 23242369 10.1038/ng.2484

[b12] Riedelsheimer C. *et al.* Genome-wide association mapping of leaf metabolic profiles for dissecting complex traits in maize. Proc. Natl. Acad. Sci. USA 109, 8872–8877 (2012). 22615396 10.1073/pnas.1120813109 PMC3384205

[b13] Riedelsheimer C. *et al.* Genomic and metabolic prediction of complex heterotic traits in hybrid maize. Nat. Genet. 44, 217–220 (2012). 22246502 10.1038/ng.1033

[b14] Bach L. *et al.* The very-long-chain hydroxy fatty acyl-CoA dehydratase PASTICCINO2 is essential and limiting for plant development. Proc. Natl. Acad. Sci. USA 105, 14727–14731 (2008). 18799749 10.1073/pnas.0805089105 PMC2567193

[b15] Spiegel S. & Milstien S. Sphingosine-1-phosphate: an enigmatic signalling lipid. Nat. Rev. Mol. Cell Bio. 4, 397–407 (2003). 12728273 10.1038/nrm1103

[b16] Fyrst H. & Saba J. D. An update on sphingosine-1-phosphate and other sphingolipid mediators. Nat. Chem. Biol. 6, 489–497 (2010). 20559316 10.1038/nchembio.392 PMC3001344

[b17] Winter E. & Ponting C. P. TRAM, LAG1 and CLN8: members of a novel family of lipid-sensing domains? Trends Biochem. Sci. 27, 381–383 (2002). 12151215 10.1016/s0968-0004(02)02154-0

[b18] Aharoni A., Dixit S. & Jetter R. The SHINE clade of AP2 domain transcription factors activates wax biosynthesis, alters cuticle properties, and confers drought tolerance when overexpressed in Arabidopsis. Plant Cell 16, 2463–2480 (2004). 15319479 10.1105/tpc.104.022897 PMC520946

[b19] Taketa S. *et al.* Barley grain with adhering hulls is controlled by an ERF family transcription factor gene regulating a lipid biosynthesis pathway. Proc. Natl. Acad. Sci. USA 105, 4062–4067 (2008). 18316719 10.1073/pnas.0711034105 PMC2268812

[b20] Kader J. C. Lipid-transfer proteins in plants. Annu. Rev. Plant Physiol. Plant. Mol. Biol. 47, 627–654 (1996). 15012303 10.1146/annurev.arplant.47.1.627

[b21] Bakan B. *et al.* Specific adduction of plant lipid transfer protein by an allene oxide generated by 9-lipoxygenase and allene oxide synthase. J. Biol. Chem. 281, 38981–38988 (2006). 17046828 10.1074/jbc.M608580200

[b22] Broun P., Poindexter P., Osborne E., Jiang C. Z. & Riechmann J. L. WIN1, a transcriptional activator of epidermal wax accumulation in Arabidopsis. Proc. Natl. Acad. Sci. USA 101, 4706–4711 (2004). 15070782 10.1073/pnas.0305574101 PMC384811

[b23] Shi J. X. *et al.* SHINE transcription factors act redundantly to pattern the archetypal surface of Arabidopsis flower organs. PLoS Genet 7, e1001388 (2011). 21637781 10.1371/journal.pgen.1001388 PMC3102738

[b24] Cernac A. & Benning C. WRINKLED1 encodes an AP2/EREB domain protein involved in the control of storage compound biosynthesis in Arabidopsis. Plant J. 40, 575–585 (2004). 15500472 10.1111/j.1365-313X.2004.02235.x

[b25] Masaki T. *et al.* ACTIVATOR of Spo^min^::LUC1/WRINKLED1 of a *Arabidopsis thaliana* transactivates sugar-inducible promoters. Plant Cell Physiol 46, 547–556 (2005). 15753106 10.1093/pcp/pci072

[b26] Baud S. *et al.* WRINKLED1 specifies the regulatory action of LEAFY COTYLEDON2 towards fatty acid metabolism during seed maturation in Arabidopsis. Plant J. 50, 825–838 (2007). 17419836 10.1111/j.1365-313X.2007.03092.x

[b27] Haslam T. M., Mañas Fernández A., Zhao L. & Kunst L. Arabidopsis ECERIFERUM2 is a component of the fatty acid elongation machinery required for fatty acid extension to exceptional lengths. Plant Physiol. 160, 1164–1174 (2012). 22930748 10.1104/pp.112.201640 PMC3490600

[b28] Marrs K. A. The functions and regulation of Glutathione S-Transferases in plants. Annu. Rev. Plant Physiol. Plant Mol. Biol. 47, 127–158 (1996). 15012285 10.1146/annurev.arplant.47.1.127

[b29] Deryabin A. N. *et al.* Influence of yeast-derived invertase gene expression in potato plants on membrane lipid peroxidation at low temperature. J. Ther. Bio. 30, 73–77 (2005).

[b30] Slack C. R., Roughan P. G. & Balasingham N. Labeling of Glycerolipids in Cotyledons of Developing Oilseeds by [1-C-14]Acetate and [2-H-3]Glycerol. Biochem J. 170, 421–433 (1978). 580379 10.1042/bj1700421 PMC1183910

[b31] Maoyin L., Welti R. & Wang X. Quantitative profiling of arabidopsis polar glycerolipids in response to Phosphorus starvation. Roles of Dξ1 and Dξ2 in phosphatidylcholine hydrolysis and digalactosyldiacylglycerol accumulation in phosphorus-starved plants. Plant Physiol. 142, 750–761. 16891548 10.1104/pp.106.085647 PMC1586058

[b32] Ganal M. W. A large maize (Zea mays L.) SNP genotyping array: development and germplasm genotyping, and genetic mapping to compare with the B73 reference genome. PLOS ONE 6, e28334 (2011). 22174790 10.1371/journal.pone.0028334 PMC3234264

[b33] Grieder C. *et al.* Determination of methane fermentation yield and its kinetics by near infrared spectroscopy and chemical composition in maize. JNIRS 19, 463–477 (2011).

[b34] Piepho H. P. Data transformation in statistical analysis of field trials with changing treatment variance. Agronomy J. 101, 865–869 (2009).

[b35] Astle W. & Balding D. J. Population structure and cryptic relatedness in genetic association studies. Stat. Sci. 24, 451–471 (2009).

[b36] Sillanpää M. J. Overview of techniques to account for confounding due to population stratification and cryptic relatedness in genomic data association analyses. Heredity 106, 511–519 (2011). 20628415 10.1038/hdy.2010.91 PMC3183892

[b37] Yu J. *et al.* A unified mixed-model method for association mapping that accounts for multiple levels of relatedness. Nat. Genet. 38, 203–208 (2006). 16380716 10.1038/ng1702

[b38] Storey J. D. & Tibshirani R. Statistical significance for genomewide studies. Proc. Natl. Acad. Sci. USA. 100, 9440–9445 (2003). 12883005 10.1073/pnas.1530509100 PMC170937

[b39] Aulchenko Y. S., Ripke S., Isaacs A. & van Duijn C. M. GenABEL: an R library for genome-wide association analysis. Bioinformatics 23, 1294–1296 (2007). 17384015 10.1093/bioinformatics/btm108

[b40] Sun G. *et al.* Variation explained in mixed-model association mapping. Heredity 105, 333–340 (2010). 20145669 10.1038/hdy.2010.11

[b41] Piepho H. P. Ridge regression and extensions for genomewide selection in maize. Crop Sci. 49, 1165–1176 (2009).

