# Supplementary material for: The maize leaf lipidome shows multilevel genetic control and high predictive value for agronomic traits
Source: Sci Rep. 2013 Aug 21;3:2479. doi: 10.1038/srep02479 (PMC3748857; doi:10.1038/srep02479)
Supplement: Supplementary Information [file srep02479-s1.pdf]

Supplementary Information for

The maize leaf lipidome shows multilevel genetic control and high predictive value for agronomic traits

Christian Riedelsheimer[13], Yariv Brotman[23], Michaël Méret[23], Albrecht E. Melchinger[1], Lothar Willmitzer[2*].

[1]Institute of Plant Breeding, Seed Science and Population Genetics, University of Hohenheim, Fruwirthstr. 21, 70593 Stuttgart, Germany

[2]Max-Planck Institute of Molecular Plant Physiology, Am Mühlenberg 1, 14476 Potsdam, Germany

[3]These authors contributed equally

[*]Corresponding author:

Lothar Willmitzer

Max Planck Institute of Molecular Plant Physiology

Am Mühlenberg 1

14476 Potsdam

Germany

Phone: +49 (331) 567 8202

Fax: +49 (331) 567 8201

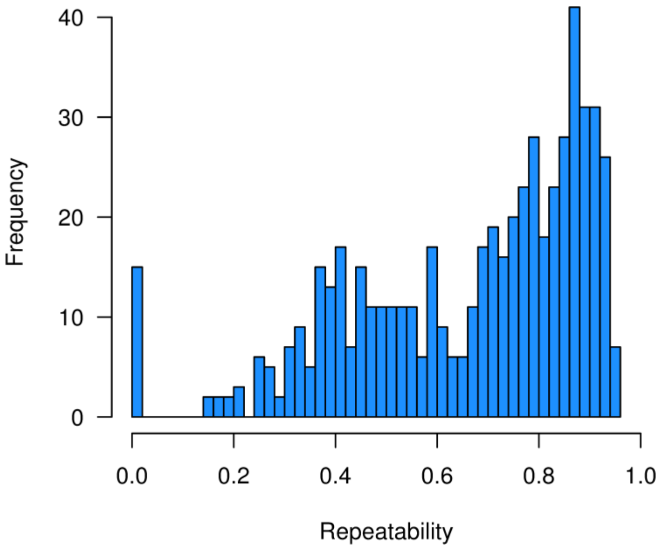

Supplementary Fig. S1 Histogram showing the repeatabilities of the measured lipids.

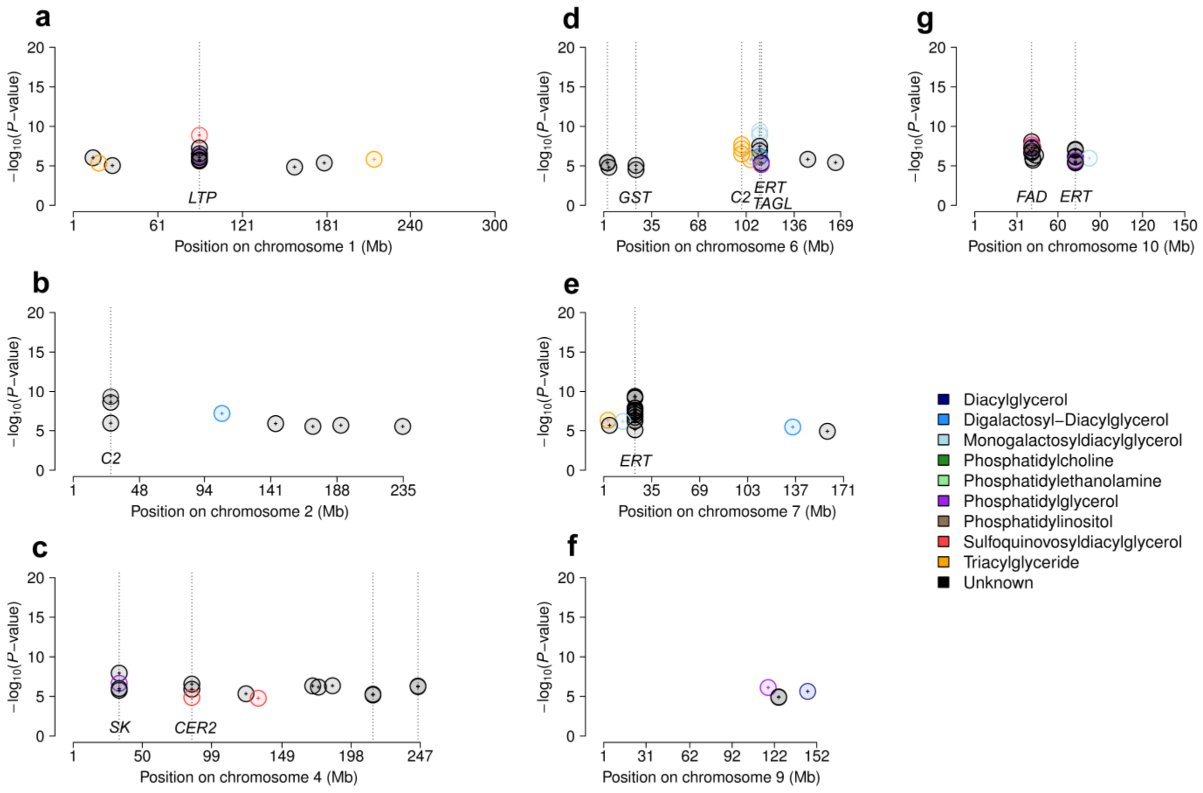

Supplementary Fig. S2 Colocalization pattern of GWA mapping hits on chromosomes 1 (**a**),2 (**b**), 4 (**c**), 6 (**d**),7 (**e**), 9 (**f**), and 10 (**g**). Center of circles refer to $-\log_{10}(P\text{-value})$ of SNP-lipid associations which were significant with FDR ≤ 0.025. Dotted lines indicate genomic positions with association signals of multiple lipids. Candidate genes with known relationships to lipid metabolism or protection against peroxidation are shown on the bottom. *LTP*, lipid transfer protein; *C2*, C2 Ca^{2+}/lipid-binding domain-containing protein; *SK*, sphingosine kinase; *CER2*, CER2 fatty acid elongase-like protein; *GST*, Glutathion-S-transferase; ERT, ethylen-responsive transcription factor; *TAGL*, triacylglycerol lipase; *FAD*, fatty acid desaturase. Details of all association hits can be found in Table S2 and candidate genes in Table 1. Colocalizations on chromosomes 3,5, and 8 can be found in **Fig. 1b-d**.

Supplementary Table S1 Descriptive details of the measured lipids.

Lipid ID	Compound	Lipid class	Intensity mean	Formula	Adduct	Measured m/z	Monoisotopic mass	Mass ppm error
866	NA	NA	318910.28	NA	NA	198.45	NA	NA
1647	NA	NA	1752720.85	NA	NA	223.39	NA	NA
1735	NA	NA	494996.90	NA	NA	226.88	NA	NA
1869	NA	NA	1686886.33	NA	NA	233.16	NA	NA
1874	NA	NA	885255.02	NA	NA	233.41	NA	NA
1879	NA	NA	467179.50	NA	NA	233.66	NA	NA
1887	NA	NA	251565.07	NA	NA	233.91	NA	NA
1901	NA	NA	270328.72	NA	NA	234.40	NA	NA
1913	NA	NA	238832.99	NA	NA	234.90	NA	NA
1995	NA	NA	4753214.19	NA	NA	238.65	NA	NA
2003	NA	NA	2560760.70	NA	NA	238.90	NA	NA
2008	NA	NA	845783.49	NA	NA	239.16	NA	NA
2013	NA	NA	258472.69	NA	NA	239.16	NA	NA
2019	NA	NA	248284.15	NA	NA	239.41	NA	NA
2020	NA	NA	142800.27	NA	NA	239.41	NA	NA
2024	NA	NA	185076.13	NA	NA	239.66	NA	NA
2026	NA	NA	224049.55	NA	NA	239.89	NA	NA
2033	NA	NA	481581.16	NA	NA	240.17	NA	NA
2036	NA	NA	167836.32	NA	NA	240.40	NA	NA
2040	NA	NA	269808.99	NA	NA	240.42	NA	NA
2074	NA	NA	253317.27	NA	NA	241.40	NA	NA
2129	NA	NA	154093.43	NA	NA	243.41	NA	NA
2223	NA	NA	275474.16	NA	NA	248.91	NA	NA
2230	NA	NA	153125.27	NA	NA	249.16	NA	NA
2937	NA	NA	160215.82	NA	NA	378.03	NA	NA
3011	NA	NA	829686.07	NA	NA	391.77	NA	NA

3012	NA	NA	819597.00	NA	NA	392.02	NA	NA
3013	NA	NA	417667.91	NA	NA	392.27	NA	NA
3014	NA	NA	146608.88	NA	NA	392.53	NA	NA
3225	NA	NA	198669.20	NA	NA	435.54	NA	NA
3226	NA	NA	252110.53	NA	NA	435.79	NA	NA
3227	NA	NA	156809.16	NA	NA	436.04	NA	NA
3268	NA	NA	472003.15	NA	NA	446.52	NA	NA
3269	NA	NA	642152.04	NA	NA	446.77	NA	NA
3270	NA	NA	580983.74	NA	NA	447.02	NA	NA
3273	NA	NA	359732.07	NA	NA	447.27	NA	NA
3351	NA	NA	169905.57	NA	NA	473.05	NA	NA
3573	NA	NA	158329.70	NA	NA	535.43	NA	NA
3576	NA	NA	221969.50	NA	NA	535.43	NA	NA
3586	NA	NA	526893.22	NA	NA	536.44	NA	NA
3599	NA	NA	590267.73	NA	NA	537.53	NA	NA
3681	NA	NA	3156432.15	NA	NA	549.49	NA	NA
3698	NA	NA	3863510.50	NA	NA	551.43	NA	NA
3700	NA	NA	2281107.78	NA	NA	551.42	NA	NA
3709	NA	NA	1055638.64	NA	NA	551.50	NA	NA
3769	NA	NA	309128.32	NA	NA	559.51	NA	NA
3809	NA	NA	155721.18	NA	NA	565.41	NA	NA
3817	NA	NA	1555451.26	NA	NA	565.57	NA	NA
3829	NA	NA	280852.16	NA	NA	567.42	NA	NA
3842	NA	NA	1242670.46	NA	NA	568.43	NA	NA
3844	NA	NA	688373.26	NA	NA	568.43	NA	NA
3847	NA	NA	674438.87	NA	NA	568.43	NA	NA
3859	NA	NA	653452.33	NA	NA	569.43	NA	NA
3884	NA	NA	6755518.82	NA	NA	571.47	NA	NA
3897	NA	NA	1465958.94	NA	NA	573.49	NA	NA

3898	NA	NA	558657.87	NA	NA	573.49	NA	NA
3899	NA	NA	447333.24	NA	NA	573.49	NA	NA
3901	NA	NA	288687.45	NA	NA	573.49	NA	NA
3907	NA	NA	305045.03	NA	NA	573.49	NA	NA
3908	NA	NA	165984.84	NA	NA	573.49	NA	NA
3909	NA	NA	197845.32	NA	NA	573.49	NA	NA
3915	NA	NA	443649.51	NA	NA	575.50	NA	NA
3917	NA	NA	179528.79	NA	NA	575.50	NA	NA
3918	NA	NA	281937.70	NA	NA	575.50	NA	NA
3920	NA	NA	151333.60	NA	NA	575.50	NA	NA
3926	NA	NA	169612.05	NA	NA	575.50	NA	NA
3941	NA	NA	249848.20	NA	NA	577.52	NA	NA
3942	NA	NA	194510.93	NA	NA	577.52	NA	NA
3978	NA	NA	366099.83	NA	NA	583.42	NA	NA
3979	NA	NA	307632.47	NA	NA	583.42	NA	NA
3981	NA	NA	189053.37	NA	NA	583.42	NA	NA
3988	NA	NA	173864.76	NA	NA	584.42	NA	NA
4001	NA	NA	2532884.99	NA	NA	585.43	NA	NA
4002	NA	NA	605509.73	NA	NA	585.43	NA	NA
4003	NA	NA	385311.18	NA	NA	585.43	NA	NA
4004	NA	NA	1070527.91	NA	NA	585.43	NA	NA
4013	DAG 34:5 (2)	Diacylglycerol	11453.31	C37H62O5	[M+H]+	587.47	586.46	3.08
4018	NA	NA	786855.28	NA	NA	587.55	NA	NA
4030	DAG 34:4 (2)	Diacylglycerol	6823.54	C37H64O5	[M+H]+	589.48	588.48	1.68
4035	NA	NA	195037.60	NA	NA	590.55	NA	NA
4046	DAG 34:3 (3)	Diacylglycerol	1872445.37	C37H66O5	[M+H]+	591.50	590.49	0.33
4047	DAG 34:3 (2)	Diacylglycerol	1246047.34	C37H66O5	[M+H]+	591.50	590.49	0.12
4067	DAG 34:2 (2)	Diacylglycerol	101290.57	C37H68O5	[M+H]+	593.51	592.51	0.56
4070	NA	NA	769803.35	NA	NA	593.60	NA	NA

4082	NA	NA	592679.59	NA	NA	595.47	NA	NA
4084	NA	NA	2238676.48	NA	NA	595.47	NA	NA
4087	DAG 34:1 (2)	Diacylglycerol	7296.90	C37H70O5	[M+H]+	595.53	594.52	1.84
4091	NA	NA	148547.65	NA	NA	595.45	NA	NA
4092	NA	NA	161163.46	NA	NA	595.47	NA	NA
4094	NA	NA	165931.96	NA	NA	595.47	NA	NA
4098	NA	NA	626386.74	NA	NA	595.59	NA	NA
4115	NA	NA	154786.91	NA	NA	597.49	NA	NA
4121	NA	NA	196085.76	NA	NA	597.49	NA	NA
4122	NA	NA	176741.65	NA	NA	597.49	NA	NA
4123	NA	NA	183552.75	NA	NA	597.49	NA	NA
4152	NA	NA	182056.86	NA	NA	599.50	NA	NA
4183	NA	NA	178528.40	NA	NA	601.43	NA	NA
4184	NA	NA	1125391.10	NA	NA	601.43	NA	NA
4185	NA	NA	362235.50	NA	NA	601.43	NA	NA
4187	NA	NA	3344492.94	NA	NA	601.43	NA	NA
4188	NA	NA	1031239.71	NA	NA	601.43	NA	NA
4189	NA	NA	2028794.06	NA	NA	601.43	NA	NA
4190	NA	NA	771627.94	NA	NA	601.43	NA	NA
4258	NA	NA	236122.59	NA	NA	608.53	NA	NA
4281	NA	NA	178036.37	NA	NA	610.62	NA	NA
4285	NA	NA	239114.62	NA	NA	611.47	NA	NA
4298	NA	NA	317801.51	NA	NA	611.56	NA	NA
4299	NA	NA	251415.97	NA	NA	611.56	NA	NA
4313	NA	NA	156763.82	NA	NA	613.48	NA	NA
4315	DAG 36:6 (3)	Diacylglycerol	6038020.53	C39H64O5	[M+H]+	613.48	612.48	0.38
4316	DAG 36:6 (2)	Diacylglycerol	20183834.79	C39H64O5	[M+H]+	613.48	612.48	0.08
4343	NA	NA	515898.08	NA	NA	615.24	NA	NA
4348	DAG 36:5 (2)	Diacylglycerol	1147835.77	C39H66O5	[M+H]+	615.50	614.49	0.22

4353	NA	NA	357802.47	NA	NA	615.58	NA	NA
4368	DAG 36:4 (2)	Diacylglycerol	366623.04	C39H68O5	[M+H]+	617.51	616.51	0.36
4385	NA	NA	521325.69	NA	NA	619.53	NA	NA
4386	DAG 36:3 (2)	Diacylglycerol	9418.57	C39H70O5	[M+H]+	619.53	618.52	1.18
4387	NA	NA	217518.26	NA	NA	619.53	NA	NA
4412	NA	NA	1068165.83	NA	NA	623.62	NA	NA
4436	NA	NA	229810.04	NA	NA	627.44	NA	NA
4461	NA	NA	161393.52	NA	NA	629.22	NA	NA
4482	DAG 36:6 (1)	Diacylglycerol	476158.39	C39H64O5	[M+NH4]+	630.51	612.48	0.17
4495	DAG 36:5 (1)	Diacylglycerol	624741.82	C39H66O5	[M+NH4]+	632.53	614.49	0.76
4509	DAG 36:4 (1)	Diacylglycerol	167457.78	C39H68O5	[M+NH4]+	634.54	616.51	1.48
4569	DAG 36:3 (1)	Diacylglycerol	5829.30	C39H70O5	[M+Na]+	641.51	618.52	0.93
4579	DAG 36:2 (1)	Diacylglycerol	5136.98	C39H72O5	[M+Na]+	643.53	620.54	1.20
4593	NA	NA	168540.35	NA	NA	645.47	NA	NA
4619	NA	NA	798774.92	NA	NA	651.65	NA	NA
4634	NA	NA	193782.64	NA	NA	654.60	NA	NA
4651	NA	NA	184563.47	NA	NA	657.43	NA	NA
4865	NA	NA	746656.53	NA	NA	682.63	NA	NA
4968	NA	NA	390582.22	NA	NA	696.54	NA	NA
5061	NA	NA	376603.47	NA	NA	708.51	NA	NA
5082	PE 34:4 (1)	Phosphatidylethanolamine	5159.15	C39H70NO8P	[M+H]+	712.49	711.48	2.01
5101	PE 34:3 (1)	Phosphatidylethanolamine	3458073.59	C39H72NO8P	[M+H]+	714.51	713.50	0.17
5103	NA	NA	1291262.59	NA	NA	714.55	NA	NA
5117	PE 34:2 (1)	Phosphatidylethanolamine	3188295.26	C39H74NO8P	[M+H]+	716.52	715.52	0.37
5135	PE 34:1 (1)	Phosphatidylethanolamine	41067.16	C39H76NO8P	[M+H]+	718.54	717.53	0.95
5160	NA	NA	220573.69	NA	NA	721.54	NA	NA
5162	NA	NA	1131822.19	NA	NA	721.50	NA	NA
5185	NA	NA	358521.82	NA	NA	723.52	NA	NA
5196	NA	NA	230369.63	NA	NA	724.57	NA	NA

5206	NA	NA	334856.33	NA	NA	726.48	NA	NA
5208	PC 32:4 (1)	Phosphatidylcholine	4415.63	C40H72NO8P	[M+H]+	726.51	725.50	0.34
5231	NA	NA	330502.65	NA	NA	730.54	NA	NA
5233	PC 32:2 (1)	Phosphatidylcholine	21165.07	C40H76NO8P	[M+H]+	730.54	729.53	0.85
5235	NA	NA	539303.13	NA	NA	731.44	NA	NA
5283	PE 36:6 (1)	Phosphatidylethanolamine	1646485.41	C41H70NO8P	[M+H]+	736.49	735.48	0.70
5285	NA	NA	315019.43	NA	NA	736.53	NA	NA
5286	NA	NA	206246.18	NA	NA	736.49	NA	NA
5304	PE 36:5 (1)	Phosphatidylethanolamine	3554120.54	C41H72NO8P	[M+H]+	738.50	737.50	2.72
5305	NA	NA	5166498.25	NA	NA	738.53	NA	NA
5323	PE 36:4 (1)	Phosphatidylethanolamine	3914600.66	C41H74NO8P	[M+H]+	740.52	739.52	0.79
5325	NA	NA	1706703.86	NA	NA	740.54	NA	NA
5344	NA	NA	231044.94	NA	NA	742.54	NA	NA
5345	PE 36:3 (1)	Phosphatidylethanolamine	92852.22	C41H76NO8P	[M+H]+	742.54	741.53	1.66
5346	NA	NA	245386.66	NA	NA	742.54	NA	NA
5347	NA	NA	742412.13	NA	NA	742.58	NA	NA
5353	NA	NA	231822.50	NA	NA	743.44	NA	NA
5355	NA	NA	3011676.53	NA	NA	743.49	NA	NA
5360	NA	NA	1225109.10	NA	NA	743.48	NA	NA
5367	NA	NA	169077.35	NA	NA	744.55	NA	NA
5370	PE 36:2 (1)	Phosphatidylethanolamine	131606.27	C41H78NO8P	[M+H]+	744.56	743.55	1.79
5376	NA	NA	590558.52	NA	NA	745.50	NA	NA
5377	NA	NA	243645.51	NA	NA	745.50	NA	NA
5382	NA	NA	292760.90	NA	NA	745.50	NA	NA
5394	NA	NA	163531.33	NA	NA	745.59	NA	NA
5395	NA	NA	167103.42	NA	NA	745.59	NA	NA
5430	NA	NA	809795.70	NA	NA	747.61	NA	NA
5447	MGDG 32:0 (1)	Monogalactosyldiacylglycerol	139328.76	C41H78O10	[M+NH4]+	748.59	730.56	0.74
5456	MGDG 32:2 (1)	Monogalactosyldiacylglycerol	5896.27	C41H74O10	[M+Na]+	749.52	726.53	1.51

5458	NA	NA	285468.87	NA	NA	749.62	NA	NA
5462	NA	NA	325918.87	NA	NA	749.62	NA	NA
5473	PC 34:6 (1)	Phosphatidylcholine	2308.47	C42H72NO8P	[M+H]+	750.51	749.50	1.96
5486	MGDG 32:1 (1)	Monogalactosyldiacylglycerol	14190.53	C41H76O10	[M+Na]+	751.53	728.54	0.58
5498	PC 34:5 (1)	Phosphatidylcholine	43291.16	C42H74NO8P	[M+H]+	752.52	751.52	1.19
5503	NA	NA	974769.76	NA	NA	752.60	NA	NA
5513	MGDG 32:0 (1)	Monogalactosyldiacylglycerol	196812.75	C41H78O10	NA	753.55	730.56	0.18
5522	PC 34:4 (1)	Phosphatidylcholine	104486.35	C42H76NO8P	[M+H]+	754.54	753.53	2.04
5523	PC 34:4 (2)	Phosphatidylcholine	786508.41	C42H76NO8P	NA	754.54	753.53	0.83
5540	NA	NA	277349.76	NA	NA	756.43	NA	NA
5543	PC 34:3 (1)	Phosphatidylcholine	41584396.49	C42H78NO8P	[M+H]+	756.55	755.55	0.74
5549	NA	NA	300888.77	NA	NA	756.68	NA	NA
5559	NA	NA	316809.89	NA	NA	757.52	NA	NA
5581	NA	NA	191231.63	NA	NA	758.45	NA	NA
5582	PC 34:2 (1)	Phosphatidylcholine	28887132.81	C42H80NO8P	[M+H]+	758.57	757.56	0.37
5584	NA	NA	202466.97	NA	NA	758.70	NA	NA
5605	PG 34:4 (2)	Phosphatidylglycerol	17915610.93	C40H71O10P	[M+NH4]+	760.51	742.48	0.70
5613	PC 34:1 (1)	Phosphatidylcholine	2700594.83	C42H82NO8P	[M+H]+	760.59	759.58	1.21
5642	NA	NA	415494.63	NA	NA	762.49	NA	NA
5645	PG 34:3 (1)	Phosphatidylglycerol	3682774.51	C40H73O10P	[M+NH4]+	762.53	744.49	0.07
5648	PG 34:3 (2)	Phosphatidylglycerol	1432513.35	C40H73O10P	[M+NH4]+	762.53	744.49	0.63
5651	NA	NA	415308.28	NA	NA	762.55	NA	NA
5656	PC 34:0 (1)	Phosphatidylcholine	778623.09	C42H84NO8P	[M+H]+	762.60	761.59	0.85
5688	PG 34:2 (1)	Phosphatidylglycerol	895935.74	C40H75O10P	[M+NH4]+	764.54	746.51	0.67
5689	NA	NA	310643.69	NA	NA	764.54	NA	NA
5700	NA	NA	414071.08	NA	NA	765.47	NA	NA
5719	NA	NA	184911.00	NA	NA	765.62	NA	NA
5726	NA	NA	249778.69	NA	NA	766.56	NA	NA
5727	PG 34:1 (1)	Phosphatidylglycerol	498932.50	C40H77O10P	[M+NH4]+	766.56	748.53	1.28

5728	NA	NA	288265.34	NA	NA	766.56	NA	NA
5754	NA	NA	351653.77	NA	NA	767.48	NA	NA
5755	NA	NA	274386.85	NA	NA	767.51	NA	NA
5776	MGDG 34:4 (1)	Monogalactosyldiacylglycerol	729940.62	C43H74O10	[M+NH4]+	768.56	750.53	0.22
5778	NA	NA	323149.19	NA	NA	768.56	NA	NA
5795	MGDG 34:6 (1)	Monogalactosyldiacylglycerol	305295.29	C43H70O10	[M+Na]+	769.49	746.50	0.03
5815	NA	NA	276682.47	NA	NA	770.57	NA	NA
5816	MGDG 34:3 (1)	Monogalactosyldiacylglycerol	11218530.68	C43H76O10	[M+NH4]+	770.58	752.54	1.16
5822	NA	NA	3086930.35	NA	NA	770.61	NA	NA
5829	MGDG 34:5 (1)	Monogalactosyldiacylglycerol	236704.59	C43H72O10	[M+Na]+	771.50	748.51	0.95
5852	NA	NA	204149.21	NA	NA	772.60	NA	NA
5855	PE 38:2 (1)	Phosphatidylethanolamine	253080.98	C43H82NO8P	[M+H]+	772.59	771.58	0.55
5857	MGDG 34:2 (1)	Monogalactosyldiacylglycerol	2882685.49	C43H78O10	[M+NH4]+	772.59	754.56	0.55
5876	NA	NA	389610.50	NA	NA	773.52	NA	NA
5892	PE 38:1 (1)	Phosphatidylethanolamine	16242.51	C43H84NO8P	[M+H]+	774.60	773.59	0.58
5902	NA	NA	294458.29	NA	NA	775.54	NA	NA
5903	NA	NA	813438.30	NA	NA	775.54	NA	NA
5944	NA	NA	231795.41	NA	NA	778.42	NA	NA
5949	PC 36:6 (1)	Phosphatidylcholine	33002066.91	C44H76NO8P	[M+H]+	778.54	777.53	0.92
5950	NA	NA	551376.96	NA	NA	778.55	NA	NA
5972	NA	NA	1022662.28	NA	NA	779.55	NA	NA
5974	MGDG 34:1 (1)	Monogalactosyldiacylglycerol	302639.96	C43H80O10	[M+Na]+	779.57	756.58	0.81
5985	NA	NA	188702.25	NA	NA	780.42	NA	NA
5986	PC 36:5 (1)	Phosphatidylcholine	28513343.90	C44H78NO8P	[M+H]+	780.55	779.55	0.38
5993	NA	NA	184047.09	NA	NA	780.70	NA	NA
5994	NA	NA	390726.13	NA	NA	780.64	NA	NA
6004	NA	NA	212773.47	NA	NA	781.56	NA	NA
6018	PG 36:7	Phosphatidylglycerol	171687.38	C42H69O10P	NA	782.50	764.46	0.44
6024	PC 36:4 (1)	Phosphatidylcholine	18691498.65	C44H80NO8P	[M+H]+	782.57	781.56	0.20

6038	NA	NA	692966.68	NA	NA	782.64	NA	NA
6039	NA	NA	452804.14	NA	NA	782.65	NA	NA
6053	NA	NA	142286.60	NA	NA	784.48	NA	NA
6060	NA	NA	927906.69	NA	NA	783.50	NA	NA
6067	NA	NA	415268.80	NA	NA	784.52	NA	NA
6068	NA	NA	3243983.73	NA	NA	784.56	NA	NA
6071	NA	NA	228911.10	NA	NA	784.55	NA	NA
6089	PC 36:3 (1)	Phosphatidylcholine	922807.44	C44H82NO8P	[M+H]+	784.58	783.58	0.47
6090	PC 36:3 (2)	Phosphatidylcholine	5083790.97	C44H82NO8P	[M+H]+	784.59	783.58	0.47
6093	NA	NA	162934.71	NA	NA	784.58	NA	NA
6097	NA	NA	186361.09	NA	NA	784.61	NA	NA
6101	NA	NA	154256.51	NA	NA	784.66	NA	NA
6102	NA	NA	577788.46	NA	NA	784.66	NA	NA
6106	NA	NA	343396.15	NA	NA	784.66	NA	NA
6109	NA	NA	258474.28	NA	NA	784.66	NA	NA
6144	NA	NA	385033.99	NA	NA	786.49	NA	NA
6151	NA	NA	778375.21	NA	NA	786.57	NA	NA
6158	PC 36:2 (1)	Phosphatidylcholine	3515651.59	C44H84NO8P	[M+H]+	786.60	785.59	0.11
6180	NA	NA	615117.60	NA	NA	787.60	NA	NA
6196	NA	NA	1515510.31	NA	NA	788.54	NA	NA
6204	NA	NA	514655.25	NA	NA	788.63	NA	NA
6206	PC 36:1 (1)	Phosphatidylcholine	194243.53	C44H86NO8P	[M+H]+	788.62	787.61	1.80
6226	NA	NA	475027.52	NA	NA	789.50	NA	NA
6234	NA	NA	242859.55	NA	NA	790.54	NA	NA
6235	MGDG 36:7 (2)	Monogalactosyldiacylglycerol	582226.33	C45H72O10	NA	790.55	772.51	0.63
6243	NA	NA	316634.93	NA	NA	789.55	NA	NA
6262	NA	NA	207978.39	NA	NA	791.55	NA	NA
6265	NA	NA	301723.17	NA	NA	791.51	NA	NA
6279	NA	NA	311139.15	NA	NA	792.56	NA	NA

6281	NA	NA	1064938.55	NA	NA	792.42	NA	NA
6282	MGDG 36:6 (1)	Monogalactosyldiacylglycerol	162157922.70	C45H74O10	[M+NH4]+	792.56	774.53	0.94
6283	NA	NA	1052009.91	NA	NA	792.70	NA	NA
6289	NA	NA	1638755.09	NA	NA	792.60	NA	NA
6304	NA	NA	266723.74	NA	NA	793.52	NA	NA
6317	NA	NA	166935.23	NA	NA	794.31	NA	NA
6318	NA	NA	215650.83	NA	NA	794.49	NA	NA
6319	NA	NA	192589.61	NA	NA	794.50	NA	NA
6320	NA	NA	170334.39	NA	NA	794.54	NA	NA
6323	MGDG 36:5 (1)	Monogalactosyldiacylglycerol	10707568.44	C45H76O10	[M+NH4]+	794.58	776.54	1.28
6348	NA	NA	153253.99	NA	NA	795.50	NA	NA
6351	NA	NA	579537.15	NA	NA	795.50	NA	NA
6352	NA	NA	379932.89	NA	NA	795.53	NA	NA
6373	MGDG 36:4 (1)	Monogalactosyldiacylglycerol	4004948.74	C45H78O10	[M+NH4]+	796.59	778.56	0.23
6399	MGDG 36:6 (2)	Monogalactosyldiacylglycerol	264664.11	C45H74O10	[M+Na]+	797.52	774.53	0.27
6413	NA	NA	207094.97	NA	NA	798.61	NA	NA
6424	MGDG 36:3 (2)	Monogalactosyldiacylglycerol	2101669.99	C45H80O10	[M+NH4]+	798.61	780.58	0.57
6432	NA	NA	1258326.81	NA	NA	798.65	NA	NA
6453	MGDG 36:5 (2)	Monogalactosyldiacylglycerol	623516.52	C45H76O10	[M+Na]+	799.53	776.54	1.46
6485	MGDG 36:2 (1)	Monogalactosyldiacylglycerol	243230.57	C45H82O10	[M+NH4]+	800.62	782.59	4.79
6501	NA	NA	3997256.16	NA	NA	801.54	NA	NA
6530	NA	NA	574111.16	NA	NA	803.55	NA	NA
6536	MGDG 36:3 (1)	Monogalactosyldiacylglycerol	210407.72	C45H80O10	[M+Na]+	803.56	780.58	0.25
6538	NA	NA	1901288.86	NA	NA	803.56	NA	NA
6558	NA	NA	271792.52	NA	NA	804.54	NA	NA
6585	NA	NA	179781.94	NA	NA	805.58	NA	NA
6600	NA	NA	335653.45	NA	NA	806.54	NA	NA
6604	NA	NA	1128741.99	NA	NA	806.54	NA	NA
6610	PC 38:6 (1)	Phosphatidylcholine	617677.07	C46H80NO8P	[M+H]+	806.57	805.56	0.56

6638	MGDG 36:1 (1)	Monogalactosyldiacylglycerol	30186.08	C45H84O10	[M+Na]+	807.59	784.61	2.46
6649	NA	NA	634326.58	NA	NA	808.56	NA	NA
6653	NA	NA	571859.35	NA	NA	808.56	NA	NA
6659	PC 38:5 (1)	Phosphatidylcholine	398994.35	C46H82NO8P	[M+H]+	808.59	807.58	0.15
6661	NA	NA	181077.34	NA	NA	808.58	NA	NA
6666	NA	NA	161170.62	NA	NA	808.67	NA	NA
6680	NA	NA	649460.04	NA	NA	809.59	NA	NA
6692	NA	NA	449321.00	NA	NA	810.58	NA	NA
6697	PC 38:4 (1)	Phosphatidylcholine	212418.75	C46H84NO8P	[M+H]+	810.60	809.59	3.34
6699	NA	NA	335695.50	NA	NA	810.61	NA	NA
6716	NA	NA	413583.54	NA	NA	811.49	NA	NA
6719	NA	NA	765048.93	NA	NA	811.50	NA	NA
6731	NA	NA	2395237.65	NA	NA	811.60	NA	NA
6749	NA	NA	283091.30	NA	NA	812.59	NA	NA
6750	NA	NA	7263479.83	NA	NA	812.55	NA	NA
6755	PC 38:3 (1)	Phosphatidylcholine	89706.87	C46H86NO8P	[M+H]+	812.62	811.61	1.07
6756	PC 38:3 (2)	Phosphatidylcholine	236496.43	C46H86NO8P	[M+H]+	812.62	811.61	1.44
6769	NA	NA	420748.62	NA	NA	812.66	NA	NA
6776	NA	NA	1195051.38	NA	NA	813.51	NA	NA
6778	NA	NA	463863.99	NA	NA	813.51	NA	NA
6779	NA	NA	232434.62	NA	NA	813.51	NA	NA
6788	NA	NA	751161.60	NA	NA	813.62	NA	NA
6798	NA	NA	245723.37	NA	NA	814.52	NA	NA
6801	NA	NA	3553968.83	NA	NA	814.61	NA	NA
6807	NA	NA	206889.00	NA	NA	814.60	NA	NA
6810	PC 38:2 (2)	Phosphatidylcholine	165254.41	C46H88NO8P	[M+H]+	814.63	813.62	0.58
6822	NA	NA	251785.01	NA	NA	815.51	NA	NA
6853	NA	NA	2233776.68	NA	NA	816.62	NA	NA
6857	NA	NA	1144095.57	NA	NA	816.66	NA	NA

6859	PC 38:1 (2)	Phosphatidylcholine	10308.90	C46H90NO8P	[M+H]+	816.65	815.64	0.85
6860	TAG 48:4 (1)	Triacylglyceride	10235.56	C51H90O6	[M+NH4]+	816.71	798.67	2.26
6867	NA	NA	370231.13	NA	NA	817.51	NA	NA
6872	NA	NA	250096.57	NA	NA	817.52	NA	NA
6895	TAG 48:3 (1)	Triacylglyceride	64876.22	C51H92O6	[M+NH4]+	818.72	800.69	1.55
6903	NA	NA	221066.86	NA	NA	819.54	NA	NA
6924	NA	NA	629906.12	NA	NA	820.59	NA	NA
6935	NA	NA	1109133.80	NA	NA	820.63	NA	NA
6940	NA	NA	223833.66	NA	NA	820.75	NA	NA
6971	NA	NA	270219.04	NA	NA	822.61	NA	NA
6984	TAG 48:1 (1)	Triacylglyceride	37394.72	C51H96O6	[M+NH4]+	822.75	804.72	1.34
7015	NA	NA	1174780.40	NA	NA	824.55	NA	NA
7023	NA	NA	242492.48	NA	NA	824.63	NA	NA
7035	TAG 48:0 (1)	Triacylglyceride	25573.57	C51H98O6	[M+NH4]+	824.77	806.74	1.97
7043	MGDG 38:6 (1)	Monogalactosyldiacylglycerol	1177701.91	C47H78O10	[M+Na]+	825.55	802.56	0.20
7058	NA	NA	230826.80	NA	NA	826.52	NA	NA
7064	NA	NA	238995.35	NA	NA	826.60	NA	NA
7080	NA	NA	543283.20	NA	NA	826.68	NA	NA
7096	MGDG 38:5 (1)	Monogalactosyldiacylglycerol	233752.14	C47H80O10	[M+Na]+	827.56	804.58	4.70
7108	NA	NA	355063.12	NA	NA	828.53	NA	NA
7119	NA	NA	275287.60	NA	NA	828.67	NA	NA
7134	NA	NA	182096.81	NA	NA	829.51	NA	NA
7136	NA	NA	913959.52	NA	NA	829.51	NA	NA
7163	NA	NA	300249.97	NA	NA	830.67	NA	NA
7171	NA	NA	480560.34	NA	NA	830.78	NA	NA
7180	NA	NA	240424.01	NA	NA	831.57	NA	NA
7181	NA	NA	366822.75	NA	NA	831.57	NA	NA
7198	SQDG 34:4	Sulfoquinovosyldiacylglycerol	161679.82	C43H74O12S	NA	832.53	814.49	1.54
7216	NA	NA	4421469.24	NA	NA	833.59	NA	NA

7239	SQDG 34:3 (1)	Sulfoquinovosyldiacylglycerol	16766371.60	C43H76O12S	[M+NH4]+	834.54	816.51	0.91
7261	NA	NA	2222835.77	NA	NA	835.60	NA	NA
7289	NA	NA	1952276.09	NA	NA	836.59	NA	NA
7290	SQDG 34:2 (1)	Sulfoquinovosyldiacylglycerol	1809383.45	C43H78O12S	[M+NH4]+	836.56	818.52	0.28
7314	NA	NA	1582623.04	NA	NA	837.62	NA	NA
7332	NA	NA	1014203.02	NA	NA	838.61	NA	NA
7338	NA	NA	396119.60	NA	NA	838.64	NA	NA
7353	NA	NA	752618.80	NA	NA	839.63	NA	NA
7355	NA	NA	140843.75	NA	NA	839.63	NA	NA
7380	NA	NA	1579263.15	NA	NA	840.62	NA	NA
7382	NA	NA	219812.59	NA	NA	840.59	NA	NA
7394	TAG 50:6 (1)	Triacylglyceride	72185.33	C53H90O6	[M+NH4]+	840.71	822.67	3.57
7402	NA	NA	167911.61	NA	NA	841.52	NA	NA
7411	NA	NA	186343.97	NA	NA	841.68	NA	NA
7429	NA	NA	581382.32	NA	NA	842.64	NA	NA
7440	TAG 50:5 (1)	Triacylglyceride	85280.65	C53H92O6	[M+NH4]+	842.72	824.69	1.43
7459	NA	NA	312099.94	NA	NA	844.65	NA	NA
7462	NA	NA	3941607.25	NA	NA	844.69	NA	NA
7465	TAG 50:4 (1)	Triacylglyceride	82388.46	C53H94O6	[M+NH4]+	844.74	826.71	1.40
7503	NA	NA	489709.05	NA	NA	846.77	NA	NA
7504	TAG 50:3 (1)	Triacylglyceride	4904442.40	C53H96O6	[M+NH4]+	846.76	828.72	0.86
7531	NA	NA	287233.74	NA	NA	848.55	NA	NA
7548	NA	NA	550171.65	NA	NA	848.66	NA	NA
7558	TAG 50:2 (1)	Triacylglyceride	2293785.08	C53H98O6	[M+NH4]+	848.77	830.74	2.13
7562	NA	NA	175681.65	NA	NA	849.58	NA	NA
7573	NA	NA	693498.95	NA	NA	850.55	NA	NA
7574	NA	NA	386204.36	NA	NA	850.60	NA	NA
7603	TAG 50:1 (1)	Triacylglyceride	500599.26	C53H100O6	[M+NH4]+	850.79	832.75	1.23
7613	NA	NA	258241.69	NA	NA	851.73	NA	NA

7618	PI 34:2 (1)	Phosphatidylinositol	426336.76	C43H79O13P	[M+NH4]+	852.56	834.53	0.26
7622	NA	NA	249880.75	NA	NA	852.65	NA	NA
7627	TAG 50:0 (1)	Triacylglyceride	16868.31	C53H102O6	[M+NH4]+	852.80	834.77	2.20
7693	SQDG 36:6 (1)	Sulfoquinovosyldiacylglycerol	14352426.23	C45H74O12S	[M+NH4]+	856.52	838.49	0.22
7733	SQDG 36:5 (1)	Sulfoquinovosyldiacylglycerol	3651565.29	C45H76O12S	[M+NH4]+	858.54	840.51	0.54
7739	NA	NA	218226.05	NA	NA	858.70	NA	NA
7742	NA	NA	260417.56	NA	NA	858.81	NA	NA
7767	SQDG 36:4 (1)	Sulfoquinovosyldiacylglycerol	479927.43	C45H78O12S	[M+NH4]+	860.56	842.52	0.79
7769	NA	NA	183282.82	NA	NA	860.54	NA	NA
7791	NA	NA	206220.21	NA	NA	861.62	NA	NA
7809	SQDG 36:3 (1)	Sulfoquinovosyldiacylglycerol	1141501.96	C45H80O12S	[M+NH4]+	862.57	844.54	0.58
7829	NA	NA	400355.14	NA	NA	863.50	NA	NA
7862	NA	NA	469051.54	NA	NA	864.63	NA	NA
7870	SQDG 38:7 (1)	Sulfoquinovosyldiacylglycerol	96686.24	C47H76O12S	[M+H]+	865.51	864.51	0.25
7895	NA	NA	1780252.43	NA	NA	866.67	NA	NA
7896	TAG 52:7 (1)	Triacylglyceride	61928.37	C55H92O6	[M+NH4]+	866.72	848.69	1.82
7900	SQDG 38:6 (1)	Sulfoquinovosyldiacylglycerol	9787.18	C47H78O12S	[M+H]+	867.53	866.52	2.45
7914	NA	NA	172643.55	NA	NA	867.68	NA	NA
7927	NA	NA	164426.78	NA	NA	868.68	NA	NA
7930	TAG 52:6 (1)	Triacylglyceride	10503836.94	C55H94O6	[M+NH4]+	868.74	850.71	0.03
7965	NA	NA	222927.33	NA	NA	870.70	NA	NA
7969	TAG 52:5 (1)	Triacylglyceride	10713794.06	C55H96O6	[M+NH4]+	870.75	852.72	0.07
7988	NA	NA	154255.22	NA	NA	871.42	NA	NA
7989	NA	NA	23356024.97	NA	NA	871.57	NA	NA
7990	NA	NA	164574.49	NA	NA	871.73	NA	NA
8010	NA	NA	584553.18	NA	NA	872.72	NA	NA
8017	TAG 52:4 (1)	Triacylglyceride	5137275.07	C55H98O6	[M+NH4]+	872.77	854.74	0.11
8045	PI 36:5 (1)	Phosphatidylinositol	106414.89	C45H77O13P	[M+NH4]+	874.54	856.51	0.56
8057	NA	NA	664899.44	NA	NA	874.79	NA	NA

8059	TAG 52:3 (1)	Triacylglyceride	2326654.07	C55H100O6	[M+NH4]+	874.79	856.75	0.85
8080	NA	NA	223981.10	NA	NA	875.80	NA	NA
8084	PI 36:4 (1)	Phosphatidylinositol	41358.17	C45H79O13P	[M+NH4]+	876.56	858.53	1.49
8091	TAG 52:2 (1)	Triacylglyceride	1022931.41	C55H102O6	[M+NH4]+	876.80	858.77	1.44
8117	TAG 52:1 (1)	Triacylglyceride	94926.61	C55H104O6	[M+NH4]+	878.82	860.78	1.90
8140	NA	NA	158265.19	NA	NA	880.69	NA	NA
8146	TAG 52:0 (1)	Triacylglyceride	15276.82	C55H106O6	[M+NH4]+	880.83	862.80	2.01
8188	NA	NA	166900.47	NA	NA	884.56	NA	NA
8212	NA	NA	809380.22	NA	NA	885.55	NA	NA
8227	NA	NA	177784.82	NA	NA	886.62	NA	NA
8254	NA	NA	316985.35	NA	NA	887.80	NA	NA
8280	MGDG 42:2	Monogalactosyldiacylglycerol	168433.68	C51H94O10	NA	889.67	866.68	0.99
8284	NA	NA	368592.10	NA	NA	889.67	NA	NA
8298	TAG 54:9 (1)	Triacylglyceride	4221541.10	C57H92O6	[M+NH4]+	890.72	872.69	0.74
8315	NA	NA	181959.39	NA	NA	891.53	NA	NA
8327	NA	NA	312089.81	NA	NA	891.68	NA	NA
8342	NA	NA	4271824.79	NA	NA	892.53	NA	NA
8345	NA	NA	186825.72	NA	NA	893.26	NA	NA
8346	NA	NA	236834.30	NA	NA	893.40	NA	NA
8347	NA	NA	33606428.95	NA	NA	893.54	NA	NA
8355	NA	NA	1164973.45	NA	NA	893.54	NA	NA
8356	NA	NA	1079709.46	NA	NA	893.55	NA	NA
8363	TAG 54:8 (1)	Triacylglyceride	7565873.06	C57H94O6	[M+NH4]+	892.74	874.71	0.23
8373	NA	NA	171686.11	NA	NA	892.83	NA	NA
8377	NA	NA	191446.28	NA	NA	894.39	NA	NA
8379	NA	NA	426788.56	NA	NA	894.70	NA	NA
8383	TAG 54:7 (1)	Triacylglyceride	6383190.54	C57H96O6	[M+NH4]+	894.75	876.72	0.14
8412	TAG 54:6 (1)	Triacylglyceride	4109897.11	C57H98O6	[M+NH4]+	896.77	878.74	0.18
8432	TAG 54:5 (1)	Triacylglyceride	2628237.04	C57H100O6	[M+NH4]+	898.79	880.75	0.08

8460	TAG 54:4 (1)	Triacylglyceride	985250.85	C57H102O6	[M+NH4]+	900.80	882.77	0.66
8492	NA	NA	246657.91	NA	NA	902.74	NA	NA
8495	NA	NA	150563.97	NA	NA	902.83	NA	NA
8496	TAG 54:3 (1)	Triacylglyceride	655478.46	C57H104O6	[M+NH4]+	902.82	884.78	1.04
8527	TAG 54:2 (1)	Triacylglyceride	189255.64	C57H106O6	[M+NH4]+	904.83	886.80	1.48
8554	NA	NA	391607.45	NA	NA	906.51	NA	NA
8565	TAG 54:1 (1)	Triacylglyceride	20910.64	C57H108O6	[M+NH4]+	906.85	888.81	2.19
8576	NA	NA	9469424.83	NA	NA	907.52	NA	NA
8579	NA	NA	285450.90	NA	NA	907.52	NA	NA
8580	NA	NA	307077.49	NA	NA	907.53	NA	NA
8613	TAG 54:0 (1)	Triacylglyceride	11942.44	C57H110O6	[M+NH4]+	908.87	890.83	2.29
8628	NA	NA	748450.83	NA	NA	909.76	NA	NA
8637	DGDG 32:0 (1)	Digalactosyl-Diacylglycerol	1490259.75	C47H88O15	[M+NH4]+	910.65	892.61	1.10
8654	NA	NA	339690.06	NA	NA	911.65	NA	NA
8657	NA	NA	561452.57	NA	NA	911.78	NA	NA
8678	NA	NA	372766.27	NA	NA	913.67	NA	NA
8680	NA	NA	169034.14	NA	NA	913.80	NA	NA
8694	DGDG 32:0	Digalactosyl-Diacylglycerol	1828274.95	C47H88O15	NA	915.60	892.61	0.42
8695	NA	NA	848423.51	NA	NA	915.52	NA	NA
8696	NA	NA	236709.18	NA	NA	915.52	NA	NA
8702	NA	NA	254024.98	NA	NA	915.68	NA	NA
8704	NA	NA	168742.03	NA	NA	915.83	NA	NA
8737	NA	NA	195981.51	NA	NA	918.74	NA	NA
8738	TAG 56:9 (1)	Triacylglyceride	84743.26	C59H96O6	[M+NH4]+	918.76	900.72	0.73
8763	NA	NA	249533.19	NA	NA	920.77	NA	NA
8764	TAG 56:8 (1)	Triacylglyceride	212555.62	C59H98O6	[M+NH4]+	920.77	902.74	1.36
8765	NA	NA	208314.70	NA	NA	920.86	NA	NA
8791	NA	NA	215594.65	NA	NA	922.69	NA	NA
8793	NA	NA	162985.81	NA	NA	922.79	NA	NA

8794	TAG 56:7 (1)	Triacylglyceride	248351.03	C59H100O6	[M+NH4]+	922.79	904.75	1.47
8827	TAG 56:6 (1)	Triacylglyceride	552501.04	C59H102O6	[M+NH4]+	924.80	906.77	0.91
8829	NA	NA	185734.39	NA	NA	925.53	NA	NA
8858	TAG 56:5 (1)	Triacylglyceride	438640.31	C59H104O6	[M+NH4]+	926.82	908.78	1.60
8877	TAG 56:4 (1)	Triacylglyceride	146392.08	C59H106O6	[M+NH4]+	928.83	910.80	1.24
8883	NA	NA	519267.24	NA	NA	929.50	NA	NA
8901	NA	NA	837078.57	NA	NA	930.61	NA	NA
8913	TAG 56:3 (1)	Triacylglyceride	144136.32	C59H108O6	[M+NH4]+	930.85	912.81	1.41
8935	NA	NA	902950.92	NA	NA	931.75	NA	NA
8946	NA	NA	178377.59	NA	NA	932.47	NA	NA
8947	DGDG 34:3 (1)	Digalactosyl-Diacylglycerol	29815813.61	C49H86O15	[M+NH4]+	932.63	914.60	0.73
8957	NA	NA	176484.78	NA	NA	932.82	NA	NA
8961	TAG 56:2 (1)	Triacylglyceride	59549.01	C59H110O6	[M+NH4]+	932.87	914.83	2.30
8976	NA	NA	1186240.52	NA	NA	933.77	NA	NA
8988	DGDG 34:2 (1)	Digalactosyl-Diacylglycerol	8555475.72	C49H88O15	[M+NH4]+	934.65	916.61	0.17
8997	TAG 56:1 (1)	Triacylglyceride	8175.63	C59H112O6	[M+NH4]+	934.88	916.85	1.61
9002	DGDG 34:4 (1)	Digalactosyl-Diacylglycerol	479561.97	C49H84O15	[M+Na]+	935.57	912.58	1.24
9004	DGDG 34:4 (2)	Digalactosyl-Diacylglycerol	412076.90	C49H86O15	NA	935.57	912.58	0.13
9005	NA	NA	288540.17	NA	NA	935.59	NA	NA
9015	NA	NA	742175.64	NA	NA	935.78	NA	NA
9027	DGDG 34:1 (1)	Digalactosyl-Diacylglycerol	746570.33	C49H90O15	[M+NH4]+	936.66	918.63	0.39
9043	NA	NA	329853.93	NA	NA	937.80	NA	NA
9084	NA	NA	254258.30	NA	NA	940.60	NA	NA
9138	NA	NA	192865.72	NA	NA	943.61	NA	NA
9159	NA	NA	340297.11	NA	NA	945.56	NA	NA
9176	NA	NA	845010.49	NA	NA	946.65	NA	NA
9212	TAG 58:8 (1)	Triacylglyceride	16083.05	C61H102O6	[M+NH4]+	948.80	930.77	1.53
9215	NA	NA	159378.08	NA	NA	948.90	NA	NA
9220	NA	NA	198870.35	NA	NA	949.61	NA	NA

9245	NA	NA	316471.33	NA	NA	950.83	NA	NA
9256	NA	NA	1137986.83	NA	NA	951.60	NA	NA
9260	NA	NA	673818.17	NA	NA	951.59	NA	NA
9261	NA	NA	208876.83	NA	NA	951.60	NA	NA
9278	TAG 58:6 (1)	Triacylglyceride	222566.04	C61H106O6	[M+NH4]+	952.83	934.80	2.17
9280	NA	NA	291277.55	NA	NA	953.56	NA	NA
9286	NA	NA	600404.92	NA	NA	954.45	NA	NA
9287	DGDG 36:6 (1)	Digalactosyl-Diacylglycerol	86393110.45	C51H84O15	[M+NH4]+	954.61	936.58	1.13
9296	TAG 58:5 (1)	Triacylglyceride	167074.69	C61H108O6	[M+NH4]+	954.85	936.81	1.89
9300	NA	NA	342858.96	NA	NA	955.80	NA	NA
9301	NA	NA	253630.66	NA	NA	955.57	NA	NA
9312	DGDG 36:5 (1)	Digalactosyl-Diacylglycerol	4667846.19	C51H86O15	[M+NH4]+	956.63	938.60	0.33
9314	DGDG 36:5 (2)	Digalactosyl-Diacylglycerol	302144.33	C51H86O15	[M+NH4]+	956.63	938.60	0.07
9319	TAG 58:4 (1)	Triacylglyceride	48884.16	C61H110O6	[M+NH4]+	956.87	938.83	1.92
9356	DGDG 36:4 (1)	Digalactosyl-Diacylglycerol	3348375.87	C51H88O15	[M+NH4]+	958.65	940.61	0.54
9383	TAG 58:3 (1)	Triacylglyceride	100007.41	C61H112O6	[M+NH4]+	958.88	940.85	1.45
9389	NA	NA	236950.05	NA	NA	960.66	NA	NA
9392	DGDG 36:3 (2)	Digalactosyl-Diacylglycerol	8628012.05	C51H90O15	[M+NH4]+	960.66	942.63	0.57
9397	TAG 58:2 (1)	Triacylglyceride	40790.67	C61H114O6	[M+NH4]+	960.90	942.86	2.31
9467	NA	NA	985000.21	NA	NA	964.81	NA	NA
9474	NA	NA	266081.47	NA	NA	965.55	NA	NA
9475	NA	NA	4509331.81	NA	NA	965.62	NA	NA
9497	NA	NA	184704.15	NA	NA	966.83	NA	NA
9500	NA	NA	164857.77	NA	NA	966.83	NA	NA
9514	DGDG 36:2 (1)	Digalactosyl-Diacylglycerol	1128717.77	C51H92O15	[M+Na]+	967.63	944.64	0.28
9546	NA	NA	253287.58	NA	NA	969.77	NA	NA
9550	NA	NA	258668.32	NA	NA	970.61	NA	NA
9588	NA	NA	200779.70	NA	NA	973.55	NA	NA
9593	NA	NA	2761037.68	NA	NA	973.66	NA	NA

9617	NA	NA	485177.07	NA	NA	975.57	NA	NA
9618	NA	NA	155932.54	NA	NA	975.57	NA	NA
9621	NA	NA	254176.29	NA	NA	975.54	NA	NA
9624	NA	NA	499890.27	NA	NA	975.67	NA	NA
9644	NA	NA	195027.58	NA	NA	977.56	NA	NA
9665	NA	NA	257530.20	NA	NA	978.87	NA	NA
9669	NA	NA	167380.28	NA	NA	979.57	NA	NA
9692	NA	NA	158640.59	NA	NA	980.81	NA	NA
9700	NA	NA	277057.20	NA	NA	981.59	NA	NA
9706	NA	NA	428941.13	NA	NA	982.64	NA	NA
9707	DGDG 38:6 (1)	Digalactosyl-Diacylglycerol	1765417.96	C53H88O15	[M+NH4]+	982.65	964.61	0.28
9713	TAG 60:5 (1)	Triacylglyceride	158208.86	C63H112O6	[M+NH4]+	982.88	964.85	1.85
9735	TAG 60:4 (1)	Triacylglyceride	48667.94	C63H114O6	[M+NH4]+	984.90	966.86	2.06
9762	NA	NA	340026.14	NA	NA	986.61	NA	NA
9769	TAG 60:3 (1)	Triacylglyceride	29824.65	C63H116O6	[M+NH4]+	986.91	968.88	2.03
9795	TAG 60:2 (1)	Triacylglyceride	12547.56	C63H118O6	[M+NH4]+	988.93	970.89	2.19
9800	DGDG 38:5 (1)	Digalactosyl-Diacylglycerol	235863.69	C53H90O15	[M+Na]+	989.62	966.63	0.42
9814	NA	NA	342572.48	NA	NA	991.56	NA	NA
9832	NA	NA	961634.74	NA	NA	992.84	NA	NA
9847	NA	NA	149911.88	NA	NA	994.86	NA	NA
9848	NA	NA	287812.74	NA	NA	994.86	NA	NA
9853	NA	NA	4944084.46	NA	NA	995.64	NA	NA
9883	NA	NA	189227.88	NA	NA	997.80	NA	NA
9918	NA	NA	682673.50	NA	NA	1001.69	NA	NA
9959	NA	NA	383221.63	NA	NA	1005.84	NA	NA
9965	NA	NA	189801.34	NA	NA	1006.90	NA	NA
9981	NA	NA	202955.09	NA	NA	1007.85	NA	NA
9989	NA	NA	654967.86	NA	NA	1008.81	NA	NA
9990	NA	NA	331277.91	NA	NA	1008.83	NA	NA

10012	NA	NA	488401.41	NA	NA	1010.83	NA	NA
10041	NA	NA	295989.37	NA	NA	1013.76	NA	NA
10043	NA	NA	184844.00	NA	NA	1013.79	NA	NA
10057	NA	NA	179077.94	NA	NA	1015.79	NA	NA
10077	NA	NA	193422.26	NA	NA	1018.74	NA	NA
10099	NA	NA	747266.67	NA	NA	1020.88	NA	NA
10118	NA	NA	571912.89	NA	NA	1022.79	NA	NA
10122	NA	NA	316948.02	NA	NA	1022.89	NA	NA
10132	NA	NA	266544.91	NA	NA	1023.70	NA	NA
10143	NA	NA	461407.90	NA	NA	1024.80	NA	NA
10178	NA	NA	230608.16	NA	NA	1027.74	NA	NA
10191	NA	NA	195550.91	NA	NA	1029.76	NA	NA
10224	NA	NA	336436.73	NA	NA	1033.87	NA	NA
10237	NA	NA	185886.40	NA	NA	1035.89	NA	NA
10248	NA	NA	267842.17	NA	NA	1036.87	NA	NA

Supplementary Table S2 Results of the genome-wide association mapping. Shown are summaries of SNP-lipid associations significant with FDF ≤ 0.025 using a $\mathbf{Q_{10} + K}$ mixed model analysis. MAF, minor allele frequency.

Lipid ID	Repeatability	Chr.	Position	SNP	MAF	*P*-value	Explained genetic variance (%)
2013	0.84	4	174839647	PZE-104099276	0.35	6.69E-07	10.97
		6	145670499	PZE-106088022	0.44	1.48E-06	10.38
2024	0.80	1	178967893	PZE-101138093	0.19	4.29E-06	10.24
		3	173118447	PZE-103114880	0.13	4.78E-07	10.24
		4	123455381	PZE-104062998	0.03	4.61E-06	10.21
		5	175460577	PZA03716.2	0.03	4.61E-06	10.21
		6	165375581	SYN4313	0.03	3.85E-06	10.65
3576	0.91	5	200055931	SYN13142	0.35	5.64E-07	11.06
		7	5105391	SYN4767	0.04	2.12E-06	8.92
3709	0.78	1	90434722	SYN12907	0.05	1.64E-06	11.91
		10	42372079	PUT-163a-76923750-4022	0.21	1.89E-06	14.77
		10	72379858	PZE-110037962	0.08	7.62E-07	14.27
3897	0.83	10	42122081	PZE-110025815	0.06	1.07E-06	11.50
		10	72379858	PZE-110037962	0.08	4.13E-06	9.68
4313	0.69	3	173418377	PZE-103115114	0.07	3.43E-07	16.49
4368	0.76	3	173118447	PZE-103114880	0.13	7.47E-09	16.48
		9	145587710	PZE-109109300	0.39	2.31E-06	11.27
5185	0.81	1	90434722	SYN12907	0.05	1.66E-06	11.65
		10	42122081	PZE-110025815	0.06	5.05E-08	17.53
		10	72379858	PZE-110037962	0.08	1.03E-07	16.52
5325	0.81	1	90434722	SYN12907	0.05	2.21E-06	11.42
		10	42122081	PZE-110025815	0.06	4.30E-08	17.73
		10	72379858	PZE-110037962	0.08	8.08E-08	16.83
5346	0.75	1	15024790	PZE-101121459	0.03	9.47E-07	12.00
		3	143778448	PZE-103089336	0.07	4.77E-07	12.85
5376	0.84	1	90434722	SYN1290	0.05	2.42E-06	10.43
		6	112736919	SYN28807	0.07	4.26E-06	9.81
		10	41583258	PZE-110025531	0.07	1.44E-07	12.80
		10	72379858	PZE-110037962	0.08	2.18E-06	9.95
5447	0.90	5	71861230	PZE-105068954	0.15	8.14E-07	10.47
		6	111523070	SYN20374	0.03	3.94E-10	16.18
5462	0.90	5	133758422	PZE-105093162	0.33	4.97E-13	20.79
5486	0.89	7	14505509	PZE-107017173	0.08	6.94E-07	10.12
5513	0.90	5	71861230	PZE-105068954	0.15	7.65E-07	10.55
		6	111523070	SYN20374	0.03	1.29E-09	15.22
5523	0.87	8	115491648	PUT-163a-78087243-4204	0.39	1.27E-13	22.37
5645	0.84	1	90434722	SYN12907	0.05	5.97E-07	11.54
		6	112736919	SYN28807	0.07	6.58E-06	9.24
		10	41583258	PZE-110025531	0.07	3.93E-08	13.73
		10	72379858	PZE-110037962	0.08	2.25E-06	9.78

5651	0.66	4	245414643	PZE-104156615	0.10	6.04E-07	19.21
5755	0.69	4	245414643	PZE-104156615	0.10	4.96E-07	18.09
5778	0.87	1	90435505	SYN12912	0.06	8.67E-07	10.16
		4	85049647	PZE-104050138	0.05	2.78E-07	11.35
		5	86624186	PZE-105077684	0.03	6.83E-07	10.60
		6	111523070	SYN20374	0.03	3.29E-08	12.93
		8	115491765	PUT-163a-50324981-2175	0.37	1.44E-07	11.97
5876	0.88	1	90435505	SYN12912	0.06	3.00E-07	10.91
		4	85049647	PZE-104050138	0.05	1.22E-06	10.12
		5	86624186	PZE-105077684	0.03	4.97E-07	10.82
		6	111523070	SYN20374	0.03	1.29E-07	11.81
		8	115491765	PUT-163a-50324981-2175	0.37	6.06E-09	14.41
5974	0.87	6	106334896	PZE-106055850	0.12	6.67E-07	11.15
6004	0.68	10	72379858	PZE-110037962	0.08	6.33E-07	15.43
6018	0.87	4	33567688	ZM008854-0420	0.02	2.21E-07	11.44
		9	117591396	PZE-109075210	0.03	7.58E-07	10.61
6109	0.88	5	133758422	PZE-105093162	0.33	3.32E-11	18.27
6151	0.74	8	17434337	PZB01977.4	0.20	1.43E-07	13.54
6204	0.82	2	144485924	PZE-102112760	0.25	1.29E-06	12.43
6235	0.86	4	33567688	ZM008854-0420	0.02	9.44E-07	10.25
		8	115491765	PUT-163a-50324981-2175	0.37	1.33E-06	10.38
		10	41362464	SYN12445	0.06	7.43E-08	12.74
6351	0.87	4	33567688	ZM008854-0420	0.02	1.02E-06	9.93
		8	115491765	PUT-163a-50324981-2175	0.37	3.31E-07	11.19
		10	41362464	SYN12445	0.06	2.13E-07	11.48
6352	0.72	4	33567688	ZM008854-0420	0.02	1.15E-08	16.34
		10	41362464	SYN12445	0.06	8.90E-09	17.49
6373	0.79	3	173118447	PZE-103114880	0.13	1.66E-08	14.84
6530	0.81	6	112736919	SYN28807	0.07	6.73E-06	9.59
		10	42122081	PZE-110025815	0.06	5.66E-08	14.22
		10	72379858	PZE-110037962	0.08	2.99E-06	9.95
6604	0.88	8	16788619	PZE-108016906	0.50	3.88E-07	11.00
6638	0.81	10	82262832	PZE-110043318	0.48	1.07E-06	11.32
6653	0.88	3	138478491	SYN18807	0.23	1.25E-05	8.43
		4	213479432	PZE-104128161	0.04	5.27E-06	9.66
		8	17706101	PZE-108018447	0.15	1.06E-08	13.32
		8	169339892	PZE-108125850	0.28	1.38E-05	8.04
		9	125052653	PZE-109081255	0.14	1.30E-05	8.15
6692	0.80	8	169339892	PZE-108125850	0.28	6.13E-07	11.79
6716	0.86	8	16788619	PZE-108016906	0.50	3.30E-07	11.34
6750	0.81	6	111523070	SYN20374	0.03	6.75E-07	13.08
6779	0.87	1	157919581	PZE-101125090	0.40	1.42E-05	8.33
		3	138478491	SYN18807	0.23	8.49E-06	8.88
		4	213479432	PZE-104128161	0.04	6.63E-06	9.61
		8	17706101	PZE-108018447	0.15	1.05E-08	13.55

		8	169339892	PZE-108125850	0.28	6.13E-07	8.55
		9	125052653	PZE-109081255	0.14	1.15E-05	8.37
6860	0.79	3	204973260	PZE-103153521	0.10	5.56E-10	18.87
6940	0.84	1	28737820	PZE-101042039	0.32	9.15E-06	9.13
		2	234576549	PZE-102194180	0.31	2.96E-06	9.69
		3	68996239	PZE-103056872	0.17	1.46E-07	14.46
		7	23152493	PZE-107022749	0.10	7.78E-06	9.14
7181	0.79	4	33567688	ZM008854-0420	0.02	1.65E-06	10.79
		10	44140416	PZE-110026674	0.23	4.55E-07	12.78
7198	0.87	1	90435505	SYN12912	0.06	1.31E-09	15.61
		3	220114271	SYN6966	0.10	3.31E-07	11.11
		4	85049647	PZE-104050138	0.05	1.34E-05	8.43
		4	132186042	PZE-104067231	0.25	1.68E-05	9.00
		5	190231321	SYN38255	0.04	2.42E-05	7.74
		10	41362464	SYN12445	0.06	2.17E-08	13.62
7394	0.91	3	203875622	PZE-103152007	0.12	4.71E-07	11.40
7440	0.90	5	48391886	PZE-105053419	0.04	6.63E-07	10.93
7791	0.79	5	70954831	SYN30642	0.09	1.26E-07	14.91
8373	0.95	2	27559497	ZM008299-0359	0.22	1.13E-06	9.18
		5	66804095	PZE-105065758	0.36	8.83E-14	23.94
		6	3592683	PZE-106002606	0.12	4.30E-06	8.54
		7	159557709	PZE-107118538	0.33	1.20E-05	7.82
		8	79865059	PZE-108048129	0.40	6.99E-06	8.28
8637	0.89	6	111523070	SYN20374	0.03	7.06E-07	10.54
8765	0.92	2	27559497	ZM008299-0359	0.22	4.80E-10	15.07
		5	66804095	PZE-105065758	0.36	8.44E-18	31.37
		6	4612971	PZE-106003733	0.12	1.51E-05	7.72
		6	23858424	PZE-106013328	0.08	8.59E-06	8.32
8791	0.91	4	170780762	SYNGENTA3398	0.04	4.69E-07	11.10
8877	0.89	3	8610977	PZE-103015689	0.09	2.13E-06	9.86
		3	228429469	SYN1581	0.44	1.03E-06	10.65
		6	105019334	PZE-106054189	0.11	1.61E-06	9.95
8901	0.91	1	90435505	SYN12912	0.06	5.60E-08	11.80
		8	115491648	PUT-163a-78087243-4204	0.39	2.08E-09	14.37
		10	41362464	SYN12445	0.06	5.90E-08	11.85
8913	0.88	1	214330598	PZE-101171300	0.24	1.47E-06	10.03
		5	202623684	PZE-105152401	0.35	1.24E-06	10.30
8961	0.86	7	4035355	PZE-107005766	0.03	4.89E-07	10.60
9004	0.78	2	106409512	PZE-102096043	0.03	6.80E-08	13.36
		3	156153305	PZE-103097515	0.06	1.45E-06	11.50
		5	141450112	PZE-105096440	0.04	3.65E-07	13.55
		7	134997749	SYN17063	0.37	3.49E-06	10.68
		8	115515327	PZE-108065360	0.43	4.56E-08	14.61
9005	0.65	2	170884908	PZE-102124713	0.03	2.95E-06	12.42
		5	86624186	PZE-105077684	0.03	1.81E-06	13.41

		5	141450112	PZE-105096440	0.04	1.81E-06	15.27
		8	115515327	PZE-108065360	0.43	1.05E-06	14.91
9215	0.93	2	27559497	ZM008299-0359	0.22	2.58E-09	14.02
		5	66804095	PZE-105065758	0.36	3.06E-15	28.35
		6	3592683	PZE-106002606	0.12	3.74E-06	8.78
		6	23858424	PZE-106013328	0.08	3.06E-05	7.29
9245	0.95	3	71549490	PZE-103057332	0.11	1.51E-06	9.25
		7	23152493	PZE-107022749	0.1	2.03E-07	11.06
9296	0.95	1	19193326	PZE-101031542	0.24	4.58E-06	8.53
		3	8610977	PZE-103015689	0.09	7.49E-07	10.00
		6	98818869	PZE-106049605	0.24	3.22E-07	10.84
9319	0.93	3	8610977	PZE-103015689	0.09	7.93E-08	11.98
		3	228429469	SYN1581	0.44	7.93E-08	8.60
		6	98818869	PZE-106049605	0.24	2.07E-08	13.06
9356	0.80	3	173118447	PZE-103114880	0.13	2.05E-07	12.12
9467	0.85	3	68792482	PZE-103056787	0.18	1.04E-07	14.40
		7	23152493	PZE-107022749	0.10	3.63E-08	14.08
9500	0.91	2	190723090	PZE-102146567	0.33	2.08E-06	9.04
		5	133758422	PZE-105093162	0.33	1.36E-09	14.61
9546	0.86	3	68792482	PZE-103056787	0.18	2.56E-07	12.97
		7	23152493	PZE-107022749	0.10	4.65E-08	13.24
9665	0.95	7	23152493	PZE-107022749	0.10	6.10E-07	10.29
9735	0.93	3	8610977	PZE-103015689	0.09	8.12E-08	12.12
		3	228429469	SYN1581	0.44	1.82E-06	9.54
		6	98818869	PZE-106049605	0.24	6.92E-08	12.12
9832	0.86	3	68792482	PZE-103056787	0.18	7.68E-08	14.24
		7	23152493	PZE-107022749	0.10	8.57E-08	12.85
9848	0.89	3	71549490	PZE-103057332	0.11	2.97E-06	9.49
		7	23152493	PZE-107022749	0.10	5.47E-07	11.11
9883	0.88	3	68792482	PZE-103056787	0.18	4.89E-10	18.35
		7	23152493	PZE-107022749	0.10	3.94E-10	16.98
9959	0.85	3	68792482	PZE-103056787	0.18	1.11E-07	14.21
		7	23152493	PZE-107022749	0.10	2.60E-08	14.17
9981	0.92	3	71549490	PZE-103057332	0.11	6.48E-07	10.40
		7	23152493	PZE-107022749	0.10	9.31E-08	11.99
10057	0.93	4	184791719	SYN3148	0.47	4.47E-07	10.04
10099	0.87	3	68792482	PZE-103056787	0.18	4.48E-09	16.52
		7	23152493	PZE-107022749	0.10	1.50E-08	14.23
10122	0.90	3	68792482	PZE-103056787	0.18	1.74E-08	14.66
		7	23152493	PZE-107022749	0.10	1.43E-08	13.84
10224	0.88	3	68792482	PZE-103056787	0.18	3.94E-09	16.92
		7	23152493	PZE-107022749	0.10	6.78E-10	16.95
10237	0.91	3	68792482	PZE-103056787	0.18	5.06E-10	17.39
		7	23152493	PZE-107022749	0.10	4.64E-10	15.99
